# A proposed sustainability index for synthesis plans based on input provenance and output fate: application to academic and industrial synthesis plans for vanillin as a case study

**DOI:** 10.3762/bjoc.16.196

**Published:** 2020-09-25

**Authors:** John Andraos

**Affiliations:** 1 CareerChem, 504-1129 Don Mills Road, Toronto, ON, M3B 2W4, Canada

**Keywords:** Borda count, green chemistry, input enthalpic energy, process mass intensity, poset dominance analysis, Rowan solvent greenness index, sacrificial reagent, sustainability, sustainable chemistry

## Abstract

This paper describes a sustainability index (SI) as a quantitative measure of “sustainability” applicable to synthesis plans based on the provenance of input materials and energy sources and the fate of output waste products. The index is computed as the root-mean-square average of the following four parameters: mass fraction of valorized inputs (*F_VI_*), mass fraction of valorized outputs (*F_VO_*), mass fraction of valorized target product (*F_VP_*), and input enthalpic energy fraction arising from renewable energy sources (*F_RE_*). Valorized input materials originate from renewable, recycled, or reclaimed sources. Valorized output materials are destined for recycling or reclaiming so that they may be used in the same or other chemical processes. Valorized target product refers to that portion of the target product that is actually used for its intended purpose. Renewable energy sources are defined as originating from hydroelectric, wind, solar, geothermal, and biomass sources. The computation of SI is illustrated for 22 synthesis plans of the high commodity flavour ingredient vanillin from biofermentation, chemical synthesis, and solvent extraction processes. In addition, these plans are compared and ranked according to Borda count and poset (partially ordered set) pairwise dominance analyses using the following attributes: process mass intensity (PMI), sacrificial reagent (SR) consumption, input enthalpic energy (IEE) consumption, Rowan solvent greenness index (RSGI), and sustainability index (SI).

## Introduction

The words “sustainable” and “sustainability” are nowadays routinely used throughout common speech and the popular press, including published modern chemistry literature, when discussing topics related to pressing issues such as preservation of the environment, climate change, and resource management. However, in all of this enormous volume of information available in the popular press, corporate mission statements, and scientific literature there does not exist an agreed consensus-based and widely used *quantitative* definition of what these words actually mean. A recent article published in Chemical and Engineering News in 2019 [1] nicely highlighted the problem in the context of distinguishing the terms *green chemistry* from *sustainable chemistry*. It was noted that “the term *sustainable chemistry* has been introduced more recently and possesses countless definitions put forth by individuals, companies, trade associations, non-profit organizations, and governmental entities”. Also noted was the “key need [to come up with] a standardized approach for assessing the sustainability of chemical processes or products”, and the need for “better information on product content throughout the supply chain and more complete data on the health and environmental impacts of chemicals throughout their life-cycle” in order for “stakeholders [to] make informed decisions that compare the sustainability of various products”. A literature search on the subject of *sustainability metrics* in chemistry journals revealed a few publications that addressed the problem of quantifying and measuring what sustainability is [2–10]. Most of the discussions revolved around extended thermodynamics analysis, energy consumption, and energy resource considerations from fossil-fuel derived and renewable sources. Dutch and Belgian chemical engineers proposed an extended thermodynamic analysis considering exergy and lost work to address the sustainability potential of the chemical process industry [2–4]. Horvath and co-workers put forth the following sustainability metrics defined for biomass-based carbon chemicals using ethanol equivalent as a common basis: sustainability value of resource replacement, sustainability value of the fate of waste, and sustainability indicator [5–7]. Sikdar defined sustainability as the interplay of three domains: economic aspects, environmental aspects, and social aspects [8]. Sheldon and Sanders defined sustainability metrics for the production of chemicals from renewable biomass in terms of four criteria: material and energy efficiency, land use, and process economics [9]. Very recently, Egyptian and Lebanese scientists put forward an industrial environmental index obtained from process, environmental health and safety, and life cycle assessment metrics to assess the sustainability of industrial solvent-based processes [10].

Continuing our goal over the last decade of developing practical and easy-to-understand metrics that can be used by any chemist or chemical engineer, in this work we introduce a quantitative description of sustainability that is directly applicable to assess synthesis plans. Synthesis plans represent the heart and soul of what chemists create in the laboratory and chemical engineers scale up and optimize in the chemical plant. Hence, any chance of developing a quantitative definition of sustainability that addresses the needs and concerns already pointed out must focus on assessing the performance of synthesis plans according to some set of measurable parameters. This is a very important aspect in the evolution of scientific ideas that begin with qualitative statements often worded in fuzzy general language, but then develops into concrete statements written in the language of mathematics that make explicit what parameters need to be measured, how they are related to one another, and what outcome scenarios are predicted from them. The net result is that vagaries are removed from the discussion and hence the subject is presented in a rigorous and understandable format that is ultimately taken seriously. Since it is evident that synthesis plans begin with input resources and ultimately produce output products, it then becomes necessary to analyze both the origins of all inputs and the fate of all outputs in order to determine the sustainability potential of a given synthesis plan. In order to achieve our objective, we decided to begin from two already established main ideas. The first is the comparison of the rate of depletion of each resource (material or energy) used in a synthesis plan versus the rate of that resource’s renewal as pointed out by Horvath and co-workers [5–7]. Clearly, sustainability is possible if the rate of renewal exceeds the rate of depletion. The second is the concept of “provenance” borrowed from the authentication of objects of art as genuine by art dealers and museum curators during the selling and purchasing of them at auctions. The provenance of an object of art potentially links it to its true creator. Obtaining provenance constitutes amassing traceable hard evidence that links the original artist and his or her work via the chain of its ownership through time from its creation to the present day. Provenances are necessary to establish the authenticity of a piece of art and thus distinguish it from a forgery. The concept of provenance or tracing has also been applied in other fields including computer science, data integrity management, petrology, archaeology, seed preservation, food authenticity, and palaeontology [11]. In the context of synthesis plans, it is possible to apply the concept of provenance to the origins of all material and energy resources used in a given plan in order to distinguish whether that resource originated from a renewable or non-renewable source. By the same reasoning, it is also possible to trace the fate of all waste outputs of a given plan in order to distinguish whether they will end up as useable or non-usable waste. From this discussion it becomes obvious that the success of tracing both provenance of inputs and fate of outputs involved in a synthesis plan in order to estimate the degree of its sustainability is based entirely on the full disclosure of supply chains and end-of-life chains throughout the chemistry enterprise. Such open access and transparent information, however, necessarily exposes vulnerabilities in those chains such as privacy with respect to business-to-business dealings and general proprietary protection that may not be comfortable to accept or possible to reveal to producers, suppliers, vendors, and end users. These concerns are at the root cause of much of the vagueness and trepidation associated with the practical application of sustainability as already pointed out earlier [1]. Nevertheless, in this work we provide a framework that can be implemented to estimate the degree of sustainability of synthesis plans following these ideas once such information is made available whether internally for a privileged few or externally for all to see. With this view in mind, in the next sections we show a step-by-step development of our methodology and then apply it to the assessment of 22 synthesis plans of the high commodity flavour ingredient vanillin.

## Methodology

We begin the development of a quantitative description of sustainability applied to synthesis plans by starting with the mathematical statement of the law of mass balance given by Equation 1.

[1] $$M_{total\;inputs}=M_{total\;outputs}$$

where the *M* quantities refer to masses in grams. The left-hand side of Equation 1 will be governed by the provenance of input materials and the right-hand side will be governed by the fate of output materials. Hence, the mass of total inputs can be subdivided into two parts: mass of valorized inputs (*M_VI_*) and mass of non-valorized inputs (*M_NVI_*) according to Equation 2.

[2] $$M_{total\;inputs}=M_{VI}+M_{NVI}$$

In this formulation, valorized inputs are defined as those that are derived from sources such that their rate of renewal is greater than or equal to their rate of depletion, and non-valorized inputs are defined as those that are derived from sources where the converse rate condition is true. Specifically, inputs derived from renewable or recycled sources such as biomass, scrap metals, or retrieved byproducts from other processes are considered valorized, and inputs derived from non-renewable sources such as fossil fuels and virgin mineral ores are considered non-valorized. The definition of *F_VI_* used here extends that of renewables intensity [12]. Similarly, the mass of total outputs may be subdivided into three parts: waste mass of valorized outputs (*W_VO_*), waste mass of non-valorized outputs (*W_NVO_*), and mass of target product (*M_product_*) as shown in Equation 3.

[3] $$\begin{array}{c} M_{total\;outputs}=W_{total}+W_{product}=W_{VO}+W_{NVO}+M_{product} \end{array}$$

Valorized waste outputs are those that may be recycled or reclaimed for use in the same synthesis plan or other unrelated synthesis plans if they are sold to other chemical enterprises in the chemical commodity supply chain. These may include reaction and work-up solvents or reaction byproducts than can be chemically converted to starting materials, or byproducts that can be reclaimed for use in other chemical processes. Non-valorized waste outputs are those that will end up as “dead waste” whether or not they undergo treatment before release into the four main environmental compartments of air, water, soil, and sediment. Based on the definition of variables in Equations 1, 2, and 3 we can then define the following key parameters. The process mass intensity (PMI) [13] is defined according to Equation 4.

[4] $$PMI=\frac{M_{VI}+M_{NVI}}{M_{product}}=\frac{M_{total\;inputs}}{M_{product}}$$

The mass fractions of valorized inputs (*F_VI_*) and valorized waste outputs (*F_VO_*) are defined according to Equation 5 and Equation 6.

[5] $$F_{VI}=\frac{M_{VI}}{M_{total\;inputs}}=\frac{M_{VI}}{M_{VI}+M_{NVI}}$$

[6] $$F_{VO}=\frac{W_{VO}}{W_{VO}+W_{NVO}}$$

We can also define a mass fraction of valorized target product (*F_VP_*) according to Equation 7 that describes the proportion of target product of a synthesis plan that is actually used for its intended purpose. This takes into account the end-of-life stage of the life cycle of the target product where part of it will end up as “dead waste”.

[7] $$F_{VP}=\frac{M_{product}-M_{product}^{*}}{M_{product}}$$

where 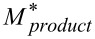 is the mass of target product that is destined to be wasted. For example, if the target product of a synthesis plan is a pharmaceutical compound a certain fraction of its manufacture will be used as intended by patients; however, there will be a remaining fraction that will be destined as non-usable waste via natural excretion by the human body and more importantly via disposal by pharmacies when the medicine passes its recommended safe expiry date. The estimation of *F_VP_* will always rely on significant assumptions and guesswork since there are no proper centralized data kept for tracking end-of-life waste of any product manufactured in the chemical industry. Hence, for the purposes of calculating SI, estimating 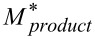 is the weakest link.

Having described the three mass fractions related to the provenance of input materials and fate of output materials, we may now examine the energy source provenance for conducting all heating and cooling operations involved in all reaction steps in a synthesis plan. We define a total input enthalpy energy, *(IEE)_total_*, as shown in Equation 8 where it is divided into renewable and non-renewable energy sources.

[8] $${\left(IEE\right)}_{total}={\left(IEE\right)}_{renewable}+{\left(IEE\right)}_{non-renewable}$$

The explicit formulation of *(IEE)_total_* is shown in Equation 9 where it is obtained as a sum of all energy consumptions as a result of heating and cooling over all input materials used in a synthesis plan above or below a reference state representing the ambient temperature and pressure conditions of 298 K and 1 atm, respectively. Temperature deviations are governed by temperature dependent heat capacity relationships for each substance, and pressure deviations are governed by volume-temperature relationships according to some specified equation of state. In practice, the contribution to IEE from temperature deviations far exceeds that from pressure deviations for reaction pressures below 100 atm. In cases where the reaction pressure exceeds 100 atm, the Redlich–Kwong equation of state formalism [14] was used in this work for computing IEE values.

[9] $$\begin{array}{l} {\left(IEE\right)}_{total}=\overset{total\;inputs}{\underset{j=1}{\sum }}{\left(IEE\right)}_{j}=\\ \overset{total\;inputs}{\underset{j=1}{\sum }}\left[moles_{j}\left({}_{+\overset{p_{rxn}}{\underset{0}{\int }}{\left[V-T{\left(\frac{\partial V}{\partial T}\right)}_{p}\right]}_{T=T_{rxn}}dp}^{\overset{T_{rxn}}{\underset{298}{\int }}C_{p,j}(T)dt+\overset{0}{\underset{1}{\int }}{\left[V-T{\left(\frac{\partial V}{\partial T}\right)}_{p}\right]}_{T=298}dp}\right)\right] \end{array}$$

Similar to the mass fractions defined in Equations 5–7, we can define an analogous input enthalpic energy fraction arising from renewable energy sources (*F_RE_*) as shown in Equation 10. This definition is similar to renewability index proposed earlier [15].

[10] $$F_{RE}=\frac{{\left(IEE\right)}_{renewable}}{{\left(IEE\right)}_{total}}$$

In the present formalism we define the following energy sources as renewable: hydroelectric, solar, wind, geothermal, and biofuels; and the following energy sources as non-renewable: coal, other fossil-fuels such as petroleum and natural gas, and nuclear. Furthermore, following recently published energy mix data [16–17] we set *F_RE_* = 0.35 for all synthesis plans that were published on or after the year 2000 and *F_RE_* = 0 for all synthesis plans that were published before 2000. We chose the year 2000 as an arbitrary boundary time frame since it marked the beginning of the 21st century when ideas of sustainability began to take root in the general societal consciousness.

Taking into account the four fractional values *F_VI_*, *F_VO_, F_VP_*, and *F_RE_* we can define an overall sustainability index (SI) which is the root-mean-square average of these four fractional quantities as shown in Equation 11.

[11] $$\begin{array}{c} SI=\frac{\sqrt{{\left(F_{VI}\right)}^{2}+{\left(F_{VO}\right)}^{2}+{\left(F_{VP}\right)}^{2}+{\left(F_{RE}\right)}^{2}}}{\sqrt{4}}\\ =\frac{1}{2}\sqrt{{\left(F_{VI}\right)}^{2}+{\left(F_{VO}\right)}^{2}+{\left(F_{VP}\right)}^{2}+{\left(F_{RE}\right)}^{2}} \end{array}$$

Since each of these fractions has values ranging between 0 and 1, then the magnitude of SI will also have a value ranging between 0 and 1. This mathematical formalism conveniently allows the writing down of a quantitative definition of sustainability applicable to synthesis plans. A given synthesis plan can therefore be said to be completely “sustainable” if the following conditions are satisfied: *F_VI_* = 1, *F_VO_* = 1*, F_VP_* = 1*, F_RE_* = 1, and SI = 1. Conversely, a given synthesis plan can be said to be completely “unsustainable”, if the following conditions are satisfied: *F_VI_* = 0, *F_VO_* = 0*, F_VP_* = 0*, F_RE_* = 0, and SI = 0.

Having this new sustainability metric in hand, we can then use it along with process mass intensity (PMI), sacrificial reagent (SR) consumption, input enthalpic energy (IEE), and Rowan solvent greenness index (RSGI) as key attributes to rank any kind of synthesis plan or chemical process. PMI and IEE have already been defined in Equations 4 and 9. SR consumption defined in Equation 12 quantifies the mass fraction of reagents whose atoms do not contribute to the chemical structure of the final target product of the synthesis plan. Following the concept of atom economy [18], this attribute is important in designing syntheses that maximize the use of all atoms in input reagents towards the final product structure while minimizing waste byproducts as a consequence of producing the intended intermediates over the course of the synthesis sequence. The RSGI [19] given in Equation 13 is a convenient metric that is used to quantify the relative environmental, toxicological, and safety-hazard impacts of solvents used in reaction, work-up, and purification procedures. It is defined using an overall solvent index (OSI) that scales between 0 and 12 spanning the benign solvent water to the non-benign solvent benzene. Equation 14 and Equation 15 show the explicit dependence of OSI on 15 physical, toxicological, and hazard parameters.

[12] $$SR=\frac{\sum mass\;sacrificial\;reagents}{\sum total\;mass\;reagents}$$

[13] $$RSGI=\sum_{i}m_{i}{\left(OSI_{12}\right)}_{i}$$

where *m_i_* is the mass of solvent *i* and OSI_12_ is defined as a normalized quantity over a set of solvents as shown in Equation 14.

[14] $${\left(OSI_{12}\right)}_{i}=12\left(\frac{OSI_{i}-OSI_{\min}}{OSI_{\max}-OSI_{\min}}\right)$$

where OSI_min_ and OSI_max_ are the minimum and maximum values of OSI for a set of solvents and OSI*_i_* for a given solvent *i* is given by Equation 15.

[15] $$\begin{array}{c} OSI_{i}=2\left(M_{OEL,i}+M_{LD50,i}+M_{LC50,i}\right)+M_{GWP,i}\\ +M_{SEP,i}+M_{ODP,i}+M_{ABP,i}+M_{BCP,i}+M_{PER,i}\\ +M_{soil,i}+M_{half\;life,i}+M_{aqua,i}+M_{Q-phrase,i}\\ +M_{SD,i}+M_{FP,i} \end{array}$$

where the metric parameters (*M*) cover occupational exposure limit (OEL, ppm), LD_50_ (ingestion toxicity, mg/kg), LC_50_ (inhalation toxicity, g m^−3^ for 4 h), global warming potential (GWP, unitless), smog formation potential (SFP, unitless), ozone depletion potential (ODP, unitless), acidity-basicity potential (ABP, unitless), bioconcentration potential (BCP, unitless), persistence potential (PER, unitless), soil sorption coefficient (soil, *K*_oc_), half-life of solvent in environment (half-life, h), aquatic toxicity to fish (aqua, mg/L for 96 h), Q-phrase potential (Q-phrase, unitless), skin dose (SD, mg), and flash point (FP, degrees K). Table 1 shows a revised and expanded formatted listing of normalized OSI values for various solvents used in the chemical industry.

**Table 1:** Revised summary of overall solvent index (OSI) for various organic solvents used in the pharmaceutical industry.

*OSI*_12_^a^		solvent

**12.000**		benzene
**10.597**		chlorobenzene
**10.350**		aniline
**10.150**		toluene
**10.130**		nitrobenzene
**10.077**		pyridine
**9.885**		triethylamine
**9.774**		*o*-xylene
**9.773**		*p*-xylene
**9.703**		*m*-xylene
**9.677**		1,2-dichlorobenzene
**9.653**		1,2-dichloroethane
**9.324**		formaldehyde
**9.048**		*n*-hexane
**8.879**		methylcyclohexane
**8.777**		cyclohexane
**8.534**		2-methyltetrahydrofuran
**8.451**		carbon tetrachloride
**8.421**		diethyl ether
**8.365**		dimethylacetamide
**8.309**		acetic anhydride
**8.090**		chloroform
**8.016**		carbon disulfide
*7.985*		tetrahydrofuran
*7.927*		acetic acid
*7.875*		*tert*-butanol
*7.852*		cyclopentyl methyl ether
*7.734*		petroleum ether
*7.727*		1,4-dioxane
*7.647*		isopropyl acetate
*7.603*		acetonitrile
*7.597*		ethyl acetate
*7.592*		*p*-*N*,*N*-dimethyltoluidine
*7.429*		*n*-heptane
*7.425*		trifluorotoluene
*7.402*		dimethylformamide
*7.365*		methyl *tert*-butyl ether
*7.323*		hexamethylphosphoric triamide
*7.278*		methyl ethyl ketone
*7.129*		dichloromethane
*7.074*		acetone
*6.966*		1-heptanol
*6.952*		1-propanol
*6.732*		methyl propionate
*6.719*		isopropanol
*6.706*		trichloroethylene
*6.644*		*n*-butanol
*6.547*		nitromethane
*6.505*		*n*-pentane
*6.108*		methyl formate
*5.905*		methyl acetate
*5.859*		ethylene glycol monomethyl ether
*5.772*		isoamyl alcohol
*5.672*		amyl acetate
*5.620*		isoamyl acetate
*5.593*		sec-butanol
*5.495*		*N*-methylpyrrolidinone
*5.426*		methanol
*5.360*		isobutyl acetate
*5.352*		anisole
*5.298*		*tert*-amylalcohol
*5.268*		cyclopentanone
*5.210*		trifluoroacetic acid
*5.110*		isopropyl ether
*5.106*		1-octanol
4.773		ethanol
4.751		ethylene glycol
4.538		thionyl chloride^b^
4.535		dimethoxymethane
4.474		isooctane
4.224		dimethyl carbonate
4.182		glycol diacetate
3.908		diglyme
3.795		sulfolane
3.679		sCO_2_
3.266		ethylene glycol dimethyl ether
3.250		triethylene glycol monomethyl ether
3.027		propylene carbonate
2.803		dimethyl sulfoxide
2.485		propylene glycol
2.233		dimethylisosorbide
0.000		water

^a^Underlined entries indicate benign performance (*OSI*_12_ ≤ 5); italicized entries indicate intermediate performance (5 < *OSI*_12_ < 8); and bold-formatted entries indicate worst performance (*OSI*_12_ ≥ 8). ^b^Missing LC_50_ (oral), LD_50_ (inhalation), and aquatic toxicity data; used as a dual reagent and solvent in industrial syntheses of acid chlorides from carboxylic acids.

In our past work [20] describing a presentation of a “standardized process green synthesis report” for chemical syntheses of pharmaceutical compounds we were able to demonstrate the Borda count [21–24] and poset (partially ordered set) pairwise dominance [25] ranking algorithms based on the four attributes PMI, SR, IEE, and RSGI. In the present work, we can now add SI as a fifth key attribute as part of those ranking algorithm analyses which takes into account sustainability potential as well as material and energy consumption and environmental and safety-hazard impacts. In order to demonstrate these ideas, we chose to examine 22 academic and industrial synthesis plans for the manufacture of 1 kg of vanillin since this high commodity flavour chemical is ideally suited to the present investigation owing to its varied methods of synthesis spanning classical chemical synthesis, biofermentation, and solvent extraction procedures from the natural source vanilla beans. We chose these particular examples from the literature since they had the most detailed experimental procedures from which the set of discussed metrics could be reliably determined and then ranked. Figure S1 found in Part 1 of Supporting Information File 1 shows all of the schemes pertaining to the 22 synthesis plans listed in alphabetical order along with temperature and pressure conditions for each reaction step. Table 2 summarizes the same alphabetized list showing the plan codes, starting materials used, and type of chemical process employed. Four plans involved biofermentation from ᴅ-glucose, ferulic acid, or isoeugenol [26–30]; five plans involved chemical synthesis from wood-derived starting materials (lignosulfonic acid liquor or sawdust) [31–37]; seven plans involved chemical synthesis from either fossil-fuel or natural product-derived starting materials (guaiacol, eugenol, isoeugenol, and 4-hydroxybenzaldehyde) [38–43]; and five plans involved solvent extraction procedures either by percolation, Soxhlet, or supercritical fluid methods using cured vanilla beans that were either whole or were cut up as starting material [44–46].

**Table 2:** Summary of plan codes, starting materials, and process types for 22 synthesis plans of vanillin.

Alphabetized list of vanillin synthesis plans	plan code	starting material	process

Borregaard synthesis	p1	lignosulfonic acid liquor	chemical synthesis
Collins chemical	p2	isoeugenol	chemical synthesis
Eilks Pt 1	p3	isoeugenol	chemical synthesis
Eilks Pt 2	p4	isoeugenol	chemical synthesis
Faith	p5	lignosulfonic acid liquor	chemical synthesis
Frost	p6	ᴅ-glucose	biofermentation
Givaudan–Roure	p7	ferulic acid	biofermentation
Haarmann and Reimer	p8	isoeugenol	biofermentation
Hibbert	p9	lignosulfonic acid liquor	chemical synthesis
Ji	p10	guaiacol	chemical synthesis
Lampman	p11	sawdust	chemical synthesis
Lesage–Meesen	p12	ferulic acid	biofermentation
Mayer	p13	eugenol	chemical synthesis
Mexican group SFE	p14	cured vanilla pods	solvent extraction
Mottern	p15	guaiacol	chemical synthesis
Ontario Paper Co.	p16	lignosulfonic acid liquor	chemical synthesis
percolation extraction cut	p17	cured vanilla pods	solvent extraction
percolation extraction whole	p18	cured vanilla pods	solvent extraction
Sorensen–Mehlum	p19	sawdust	chemical synthesis
Soxhlet extraction cut	p20	cured vanilla pods	solvent extraction
Soxhlet extraction ground	p21	cured vanilla pods	solvent extraction
Taber	p22	4-hydroxybenzaldehyde	chemical synthesis

## Results and Discussion

### Sustainability of vanillin plans

The exercise of partitioning input materials according to their provenance and output materials according to their fate is extremely challenging because it requires a completely transparent knowledge and access to the entire network supply and end-of-life chains that constitute the chemical industry enterprise. In practice, when reading experimental sections in journal publications and patents, one has limited or no access to such background knowledge even though authors may disclose the names of chemical suppliers of the starting materials they used in their own work. It is a fair comment to say that authors themselves may not know or care to know the chain of supply of those starting materials so long as they have them in hand in a sufficiently pure condition to carry out their own research agenda. In any event, the task of estimating a quantitative measure of sustainability of any given synthesis plan according to the formalism of Equation 11 will require significant assumptions to be made. For the purpose of our work, which is meant to be illustrative only in the numerical methods employed, we implemented the following assumptions in the calculation of SI for all 22 plans of vanillin:

(1) If ethanol is used as an input material then 10% of it was assumed to originate from renewable sources (i.e., biomass), if the publication is dated after 1990 since that is the approximate time frame when biofuels were made widely available in the market.

(2) Water was considered a renewable input material due to the circulating global hydrological cycle.

(3) Mineral salts, metal-derived catalysts, and all non-aqueous and non-biologically derived materials from fossil fuels or ores were considered non-renewable inputs since their rate of renewal occur on geological time scales that are several orders of magnitude longer than organism time scales.

(4) Lignosulfonic acid liquor and sawdust were considered renewable inputs since they ultimately originate from trees. Ferulic acid and ᴅ-glucose were considered renewable inputs since they originate from sugar beet biomass [47] and other cellulosic biomass feedstocks, respectively. Isoeugenol and eugenol were chosen to originate from the natural product clover oil since 80% by weight of this essential oil is eugenol [48], and isoeugenol is directly obtained from eugenol by base-catalyzed isomerization.

(5) Oxygen from air was considered a renewable input for the oxidation of lignosulfonic acid liquors since it is produced as a natural waste byproduct of photosynthesis.

(6) Spent vanilla beans were considered reusable waste since they can be composted into biomass.

(7) Supercritical carbon dioxide was considered reusable waste since it can be recycled in the same solvent extraction process via continuous pressurization and depressurization cycles.

(8) A renewable energy source contribution of 35% (*F_RE_* = 0.35) was assigned, if the publication is dated on or after the year 2000 for reasons discussed earlier.

(9) Since vanillin is mainly used as a food flavouring agent, 70% of it was assumed to be actually used in the food industry (*F_VP_* = 0.7) and 30% of it will end up as part of the food waste stream which mirrors recent statistics [49–53] that assert that about a third of all foodstuffs produced will end up wasted along supply chains and by end-of-use consumers.

**Figure 1:**
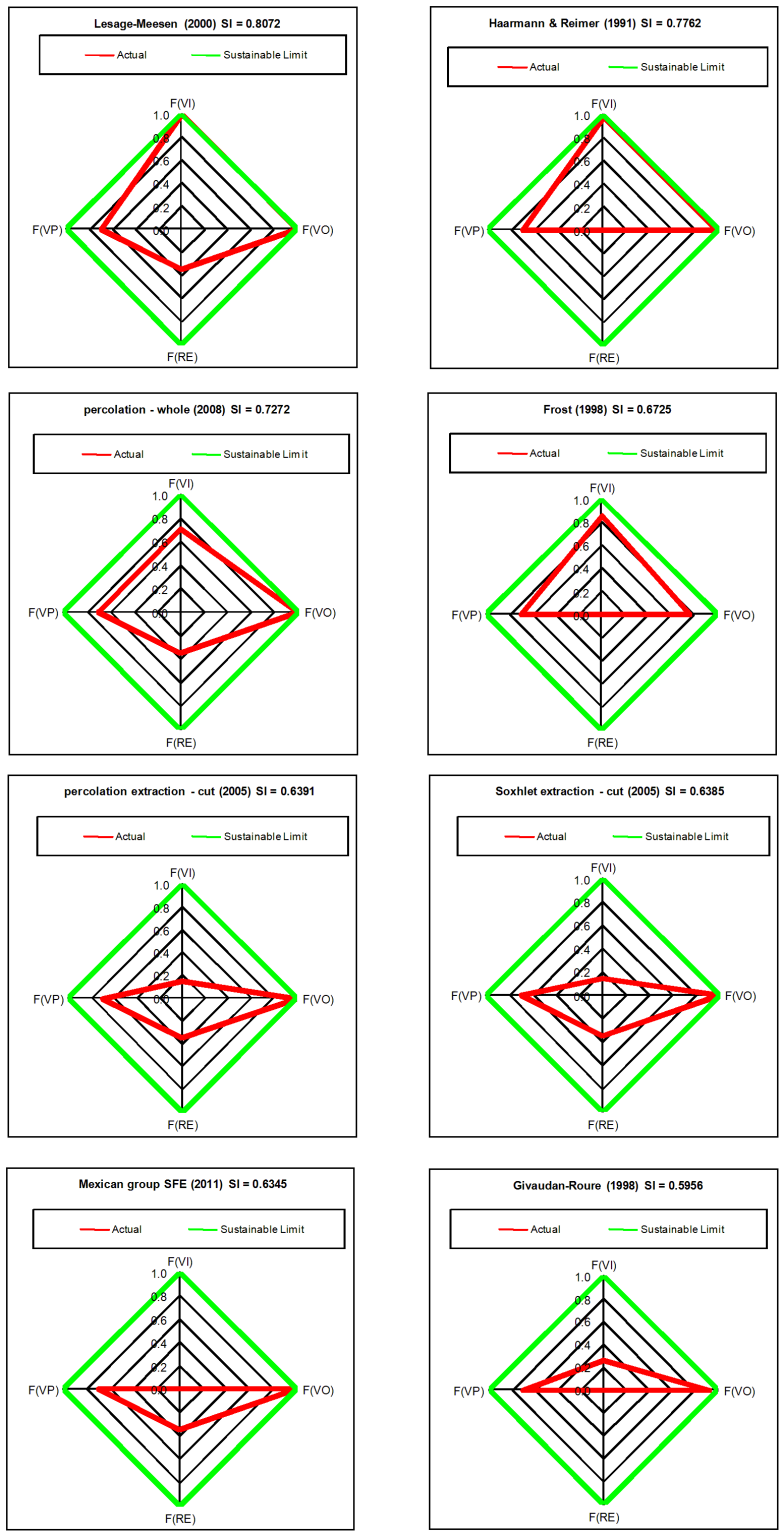
Radial diamond diagrams illustrating the sustainability index (SI) computed based on *F_VI_*, *F_VO_*, *F_VP_*, and *F_RE_* for the top eight scoring vanillin synthesis plans.

In order to facilitate computation of SI an Excel-based calculator was developed that can be used for any synthesis plan once all inputs and outputs are identified (see Supporting Information File 2). Firstly, for a given synthesis plan, all scaled masses of input and output materials are entered for the production of 1 kg of vanillin. Scaled mass data for each plan are found in Part 2 of Supporting Information File 1. Secondly, valorized input and output materials are selected according to the assumptions listed above. Finally, *F_RE_* is set to 0.35 or 0 for plans published after or before the year 2000, respectively and *F_VP_* is set at 0.7. Figure 1 shows radial diamond diagrams depicting the four fractions for the leading eight most sustainable plans representing biofermentation and solvent extraction methods. The main reason for their high SI scores is the combined high values of *F_VI_* and *F_VO_* close to unity in each case. For the solvent extraction procedures low values of *F_VI_* are found if the mass ratio of solvent to vanilla bean is very high as is the case for the percolation extraction cut, Soxhlet extraction cut, and Mexican group SFE plans. For the case of supercritical carbon dioxide used as an extraction solvent, the main drawback is that industrial carbon dioxide is classified as originating from a non-renewable mineral resource (mainly calcium carbonate) and that the mass ratio of sCO_2_ solvent to vanilla beans is 435:1. Synthesis plans based on chemical syntheses from lignosulfonic acid liquor, guaiacol, eugenol and isoeugenol have *F_VO_* values of 0 since none of their solvent and waste byproducts are reuseable. However, *F_VI_* values for these syntheses range between 0 (Eilks – Pt 2) and 0.898 (Borregaard) with an average value of 0.414 over 14 plans. With respect to renewable energy sources, only nine out of the 22 plans were assigned a value of 0.35 for *F_RE_* since their publications appeared on or after the year 2000.

Table 3 summarizes numerical results of the 5 attributes PMI, SR, IEE, RSGI, and SI for all 22 synthesis plans of vanillin. From this list we observe that chemical syntheses tend to have lower PMI values and biofermentation and solvent extraction methods tend to have higher PMI values due to higher solvent consumption in the latter group. Ten out of the 22 plans had SR values of 0 and the rest of the plans had higher values in the range 1.2 to 92, except for the Eilks – Pt 1 and Lampman plans that used isoeugenol and sawdust, respectively, as starting materials. With respect to energy consumption, the two percolation extraction procedures using cut or whole cured vanilla beans with no heating of solvents had the lowest IEE value of 0. The other plans had significant energy demands for various reasons. The methods starting from lignosulfonic acid liquors were conducted under elevated temperature and pressure conditions of 200 °C and 100 atm. The SFE method using a high amount of sCO_2_ required pressurization conditions of about 200 atm. The biofermentation methods, though they were conducted under biologically ambient conditions of 37 °C and 1 atm, had high IEE values as a result of the high mass of aqueous nutrient broths required for their operation relative to the small mass of starting materials. For example, the most sustainable Lesage–Meesen plan with the highest SI value of 0.8072 had an IEE value of 939,475 kJ/kg owing to the 1254:1 combined aqueous nutrient medium-to-substrate ratio. With respect to solvent impacts, the Givaudan–Roure, Lesage-Meesen, and Haarmann and Reimer biofermentation procedures had RSGI values of 0. The only other plan with a zero solvent impact was the improved lignosulfonate method of Borregaard carried out in aqueous solution. The traditional chemical synthesis methods had more of a solvent impact due to the use of the following solvents listed in descending order of impact according to OSI values given in Table 1: benzene (12.000), aniline (10.350), toluene (10.150), nitrobenzene (10.130), cyclohexane (8.777), diethyl ether (8.421), acetic anhydride (8.309), petroleum ether (7.734), ethyl acetate (7.597), *N,N*-dimethyltoluidine (7.592), methyl *tert*-butyl ether (7.365), dichloromethane (7.129), methanol (5.426), and ethanol (4.773). The Lampman plan had the highest overall RSGI value of 112,106 kg/kg vanillin due to the high masses of impactful solvents employed to obtain 1 kg of vanillin product, namely, nitrobenzene, diethyl ether, and cyclohexane.

**Table 3:** Summary of computed values for five attributes for 22 synthesis plans of vanillin.

Alphabetized list of vanillin synthesis plans	PMI (kg/kg)	SR (kg/kg)	IEE (kJ/kg)	RSGI (kg/kg)	SI

Borregaard synthesis	84	8.6	169046	0	0.5756
Collins chemical	38	1.2	50440	38	0.4603
Eilks Pt 1	1712	341	17309	4492	0.4866
Eilks Pt 2	147	0	2881	673	0.3907
Faith	50	0	29286	6	0.4519
Frost	15973	92	578763	16369	0.6725
Givaudan–Roure	21	0	795	0	0.5956
Haarmann and Reimer	1159	0	9388	0	0.7762
Hibbert	803	71	1005329	3768	0.4207
Ji	3843	45	325200	24670	0.3914
Lampman	32540	9845	2398980	112106	0.3676
Lesage–Meesen	19434	30	939475	0	0.8072
Mayer	26	10	20989	163	0.3704
Mexican group SFE	7513	0	1678005	27578	0.6345
Mottern	125	0	2181	277	0.4551
Ontario Paper Co.	730	32.5	688521	2101	0.4689
percolation extraction cut	831	0	0	3727	0.6391
percolation extraction whole	1028	0	0	1579	0.7272
Sorensen–Mehlum	563	29.5	653838	1952	0.4171
Soxhlet extraction cut	582	0	50219	2644	0.6385
Soxhlet extraction ground	5332	0	676694	16828	0.4246
Taber	1943	1.4	28138	12327	0.3946

### Ranking of vanillin plans

Once numerical values of PMI, SR, IEE, RSGI, and SI are available for a given set of synthesis plans to a common target product, it is possible to use some kind of ranking algorithm to identify which ones have the highest overall performances based on these five attributes. From our previous work [20] we compared and contrasted the Borda positional counting method [21–24], established in 1781 by Jean-Charles de Borda (1733–1799), and the poset pairwise dominance analysis method [25] on 6 synthesis plans for the pharmaceutical apixaban. In implementing the Borda count method we first list the plans in ascending order of PMI, SR, IEE, and RSGI so that plans having the lowest values for these attributes are ranked highest; and we list the plans in descending order of SI so that plans having the highest values of SI are ranked highest. Since in this analysis there are 22 vanillin plans to consider the top ranking plan in any given list is assigned a point value of 22 and the lowest ranking plan is assigned a point value of 1 with all other plans having intermediate points accordingly. The maximum Borda count score corresponds to the number of plans considered in the set. In cases of plans having the same numerical values for a given attribute, they are assigned the same Borda count score. For example, for the SR attribute there are 10 plans that have an SR value of 0. Hence, each of them is assigned a Borda count score of 22 and the following lower ranking plans are successively given lower values in descending order. In this case the lowest ranking SR plan is given a Borda count score of 10. Once all Borda count scores are determined for all plans for each attribute, the scores for each plan are added up and these summed scores are ranked from highest to lowest. Table 4 shows the net results of the Borda count method implemented on all 22 synthesis plans for vanillin considered. Part 3 of Supporting Information File 1 contains all of the Borda count data. We observe that the Givaudan–Roure (102) and Haarmann and Reimer (92) biofermentation routes have the highest overall ranking across the 5 attributes according to the Borda count method followed closely by the percolation extraction of whole vanilla beans (90), Faith synthesis from lignosulfonic acid liquor (87), and Mottern four-step chemical synthesis from guaiacol (87). The lowest ranking plan was found to be the Lampman synthesis from sawdust, which had the highest overall process mass intensity (PMI = 32,540 kg/kg), second highest energy consumption requirements (IEE = 2,398,980 kJ/kg), fourth-highest solvent impact ranking (RSGI = 112,106 kg/kg), and lowest sustainability index value (SI = 0.3676).

**Table 4:** Summary of Borda count results for 22 synthesis plans of vanillin.^a^

Alphabetized list of vanillin synthesis plans	PMI points	SR points	IEE points	RSGI points	SI points	overall Borda count

Borregaard synthesis	18	19	11	**22**	14	84
Collins chemical	19	21	12	20	11	83
Eilks Pt 1	8	11	17	10	13	59
Eilks Pt 2	16	**22**	19	17	3	77
Faith	20	**22**	15	21	9	87
Frost	3	12	9	8	19	51
Givaudan–Roure	**22**	**22**	21	**22**	15	**102**
Haarmann and Reimer	9	**22**	18	**22**	21	**92**
Hibbert	12	13	4	11	7	47
Ji	6	14	10	6	4	40
Lampman	1	10	2	4	1	18
Lesage–Meesen	2	16	5	**22**	**22**	67
Mayer	21	18	16	19	2	76
Mexican group SFE	4	**22**	3	5	16	50
Mottern	17	**22**	20	18	10	87
Ontario Paper Co.	13	15	7	14	12	61
percolation extraction cut	11	**22**	**22**	12	18	85
percolation extraction whole	10	**22**	**22**	16	20	90
Sorensen–Mehlum	15	17	8	15	6	61
Soxhlet extraction cut	14	**22**	13	13	17	79
Soxhlet extraction ground	5	**22**	6	7	8	48
Taber	7	20	14	9	5	55

^a^Entries highlighted in bold represent the highest scores in each of the 5 attribute categories.

In implementing the poset pairwise dominance algorithm we determine the number of pairwise attributes and the number of pairwise plan comparisons for each pairwise attribute in order to determine the overall size of the ranking exercise. Since there are 5 attributes the number of pairwise attribute comparisons is C(5,2) = 5!/((5 – 2)! 2!) = 10. The explicit list is as follows: PMI versus SR, PMI versus IEE, PMI versus RSGI, PMI versus SI, SR versus IEE, SR versus RSGI, SR versus SI, IEE versus RSGI, IEE versus SI, and RSGI versus SI. Since there are 22 synthesis plans for vanillin the number of pairwise plan comparisons is C(22,2) = 22!/((22 – 2)! 2!) = 231. Hence, there are overall 10 × 231 = 2310 pairwise comparisons that need to be made in the entire poset analysis. In general, a complete poset analysis on *K* synthesis plans to a common target product based on *m* attributes will require C(*K*, 2)*C(*m*, 2) = (*K*!/(*K* – 2)! 2!)·(*m*!/(*m* – 2)! 2!) pairwise comparisons. For a given pairwise plan comparison for a pair of attributes there are two possible outcomes: (a) a comparable pair in which plan A dominates plan B for *both* attributes X and Y; and (b) an incomparable pair in which plan A dominates plan B for attribute X and plan B dominates plan A for attribute Y. For facile visual display of the results upper triangular 22 × 22 matrices are constructed showing green-coloured entries for comparable pairs and red-coloured entries for incomparable pairs. When a comparable pair for a given pairwise attribute comparison is found the dominant plan is identified. This sequence of steps is repeated for each pairwise attribute comparison and then the number of dominant occurrences for each plan are tallied up. Part 4 of Supporting Information File 1 summarizes the ten 22 × 22 matrices and the number of dominances for each plan for each pairwise attribute comparison. Supporting Information File 3 contains an Excel template file that facilitates carrying out the tedious task of pairwise comparisons involved in the poset analysis, particularly when the number of synthesis plans under consideration is large. Table 5 summarizes the main results of the poset dominance analysis for all vanillin plans considered. We observe that plans p7 (Givaudan–Roure), p8 (Haarmann and Reimer), and p18 (percolation extraction whole vanilla beans) have the highest number of pairwise dominances of 145, 105, and 101, respectively. Plans p11 (Lampman synthesis from sawdust), p10 (Ji synthesis from guaiacol), p14 (Mexican group SFE), p9 (Hibbert synthesis from lignosulfonic acid liquor), p21 (Soxhlet extraction from ground vanilla beans), and p6 (Frost biofermentation from ᴅ-glucose) have the fewest number of dominances of 0, 18, 23, 27, 28, and 29, respectively. Table 6 and Table 7 summarize the results of the two ranking algorithms. Both methods identify the same set of overall best plans and overall worst plans with 11 out of the 22 plans having exactly the same ranking. With respect to plans having different ranking orders we find that 5 out of 22 plans have +/− 1 rank positional change, 5 out of 22 plans have +/− 2 rank positional change, and 1 plan out of 22 having a +/− 3 rank positional change. Overall, the faster Borda count method is able to quickly identify the top and bottom performing plans with certainty. The more tedious poset pairwise dominance analysis is more reliable in ranking the intermediate performing plans due to its thoroughness in considering all possible pairwise plan comparisons across all attributes considered.

**Table 5:** Summary of pairwise poset dominance analysis for 22 synthesis plans of vanillin based on 5 attribute categories.^a^

pairwise attribute comparison	number of dominances																					
	p1	p2	p3	p4	p5	p6	p7	p8	p9	p10	p11	p12	p13	p14	p15	p16	p17	p18	p19	p20	p21	p22

PMI vs SR	8	10	1	9	10	1	**12**	6	3	2	0	1	8	3	9	5	6	6	7	8	3	4
PMI vs IEE	9	10	7	13	11	8	**19**	2	3	5	0	1	14	1	9	4	9	9	6	8	3	6
PMI vs RSGI	17	16	5	12	15	1	**21**	8	7	2	0	1	15	1	14	9	7	7	11	9	2	5
PMI vs SI	10	9	4	1	7	1	**14**	7	3	1	0	1	1	1	7	5	6	6	3	7	1	2
SR vs IEE	7	8	1	12	9	1	12	12	1	3	0	2	7	3	12	2	**13**	**13**	4	9	4	8
SR vs RSGI	8	9	1	9	9	1	**11**	**11**	3	1	0	6	7	1	8	5	6	8	6	6	2	3
SR vs SI	7	5	1	2	7	2	10	**11**	1	1	0	5	1	10	6	4	9	**11**	2	10	6	3
IEE vs RSGI	9	8	6	11	10	3	**18**	15	2	1	0	3	10	1	11	3	8	11	5	6	3	5
IEE vs SI	7	5	10	2	5	7	14	15	1	1	0	3	1	1	9	3	17	**18**	1	9	2	2
RSGI vs SI	13	8	3	1	8	4	14	**18**	3	1	0	**18**	1	1	8	5	7	12	3	7	2	2

TOTALS	95	88	39	72	91	29	**145**	105	27	18	0	41	65	23	93	45	88	101	48	79	28	40

^a^Entries highlighted in bold represent the highest dominances in each of the 10 pairwise attribute comparisons.

**Table 6:** Summary of Borda count and poset dominance analysis of 22 synthesis plans for vanillin listed alphabetically.

Alphabetized list of vanillin synthesis plans	plan code	Borda count	poset dominances

Borregaard synthesis	p1	84	95
Collins chemical	p2	83	88
Eilks Pt 1	p3	59	39
Eilks Pt 2	p4	77	72
Faith	p5	87	91
Frost	p6	51	29
Givaudan–Roure	p7	102	145
Haarmann and Reimer	p8	92	105
Hibbert	p9	47	27
Ji	p10	40	18
Lampman	p11	18	0
Lesage–Meesen	p12	67	41
Mayer	p13	76	65
Mexican group SFE	p14	50	23
Mottern	p15	87	93
Ontario Paper Co.	p16	61	45
percolation extraction cut	p17	85	88
percolation extraction whole	p18	90	101
Sorensen–Mehlum	p19	61	48
Soxhlet extraction cut	p20	79	79
Soxhlet extraction ground	p21	48	28
Taber	p22	55	40

**Table 7:** Summary of Borda count and poset dominance rankings of 22 synthesis plans for vanillin.^a,b^

Borda count ranking	plan	Poset dominance ranking	plan

*102*	*p7*	*145*	*p7*
*92*	*p8*	*105*	*p8*
*90*	*p18*	*101*	*p18*

87	**p5**	95	**p1**

*87*	*p15*	*93*	*p15*

85	**p17**	91	**p5**
84	**p1**	88	**p2**
83	**p2**	88	**p17**

*79*	*p20*	*79*	*p20*
*77*	*p4*	*72*	*p4*
*76*	*p13*	*65*	*p13*

67	**p12**	48	**p19**

*61*	*p16*	*45*	*p16*

61	**p19**	41	**p12**
59	**p3**	40	**p22**
55	**p22**	39	**p3**

*51*	*p6*	*29*	*p6*

50	**p14**	28	**p21**
48	**p21**	27	**p9**
47	**p9**	23	**p14**

*40*	*p10*	*18*	*p10*
*18*	*p11*	*0*	*p11*

^a^Line separated, italicized entries represent plans having exactly the same ranking order. ^b^Bold entries represent plans having different ranking orders.

## Conclusion

We have introduced and demonstrated how a sustainability index (SI) can be computed specifically for synthesis plans based on the provenance of input materials and energy sources, and the fate of output waste materials. Reasonable and reliable estimates of SI based on provenance and fate of input and output materials respectively can only be made if full disclosure of both supply and disposal chains in the chemical enterprise exists. We note that this is a formidable challenge for the chemical community to accept and adopt in routine practice. We also note that the computation of SI will necessarily involve significant assumptions to be made in determining key parameters such as *F_RE_* and *F_VP_* and that these assumptions, in turn, will necessarily affect the ranking of synthesis plans. The nine assumptions listed for the analysis of vanillin are an illustrative example of what is entailed for the computation of SI. Other target products will require their own set of assumptions. Nevertheless, we believe that the protocols disclosed in this work are easily implementable once the necessary data are made available.

In our determination of the four fractions contributing to SI we implemented a binary approach based on whether or not a material or energy input arises from renewable or non-renewable sources; and on whether or not an output material could be recycled or reused. Specific rates of depletion versus renewal of a resource applied to inputs and specific rates of reusability versus accumulation of outputs require a complete macroscopic knowledge and connectivity of all elements pertaining to the network of all chemical processes involved in a given synthesis plan. At this time reliable estimates of these rates are not readily available to the average practicing chemist or chemical engineer in established open-access data collection databases for all commodity material resources, and so this significant limitation prevents estimation of time analyses pertaining to how long a given resource may exist under a so-called “sustainability condition”. For first generation chemical feedstocks arising from fossil fuels, the rate of finding new reserves of fossil fuel may be used as a rate of “renewal” rather than the geological rate of renewal which is several orders of magnitude lower. In any case, rates of finding new reserves of fossil fuels or mineral deposits depend on knowledge of counting existing reserves, which necessarily requires reliable databases that constantly track data annually. Such tracking is not always in industry’s best interest to disclose such information publicly for economic and political reasons. For example, providing inaccurate or incomplete information to governments and investors can leverage control of prices of crude oil, natural gas, and metals; whereas, disclosing accurate and up-to-date information can expose vulnerabilities among governments and investors that can be taken advantage of.

Furthermore, deciding where to terminate a chain of resources, i.e., which material to designate as the “starting material” for a given synthesis plan, remains a non-resolvable dilemma. What is known with certainty is that extending a starting material chain will necessarily amplify material consumption (PMI), enthalpic energy inputs (IEE), and associated environmental and safety-hazard impacts (RSGI). Hence, if the starting points of the vanillin plans in this work are changed, particularly for the chemical syntheses, then this will change the values of all of these parameters and ultimately the ranking order of the plans based on those parameters. There is also the problem of including the syntheses of all reagents and catalysts used in each reaction step involved in a given synthesis plan. Again, significant amplification of material consumption (PMI), enthalpic energy inputs (IEE), and associated environmental and safety-hazard impacts (RSGI) will result. For the case of *F_VI_* which is the ratio of mass of valorized input to mass of total input, if we were to include the synthesis route of a catalyst used in the main synthesis chain we can envisage two case scenarios for how this will impact the computation of *F_VI_* where the mass of total inputs (denominator) will obviously increase. In one case, if the mass of inputs to make the catalyst is considered valorized, then the numerator magnitude will also increase. Hence, the overall value of *F_VI_* is expected to increase which will in turn increase the value of SI. On the other hand, if the mass of inputs to make the catalyst is considered non-valorized, then the numerator magnitude does not change. Hence, the overall value of *F_VI_* is expected to decrease which will in turn decrease the value of SI. Similar trends apply for *F_VO_* and *F_RE_*. Such a scenario will have a negative impact on chemical syntheses that primarily use non-renewable materials, reagents, and catalysts. The net effect is to drive down the ranking of chemical syntheses. Chemical syntheses have a lower likelihood of achieving moderate levels of sustainability compared to ones based on biofeedstocks and biofermentation processes mainly because they utilize fossil fuel-derived materials. In any event, the task of tracing starting materials, catalysts, and reaction solvent syntheses is very tedious, especially for time-pressed chemists who wish to practice green chemistry. However, such a task can be significantly alleviated if synthesis databases of first, second, and third generation feedstocks existed where all key metrics have been worked out in advance such as PMI, SR, IEE, and RSGI. It is then possible to tap into these databases in a cassette-like manner and insert these synthesis chain extensions to the main synthesis chain of interest as needed. The creation of such databases requires significant investment in time and energy but once done it is expected that they will have far-reaching utility in the long run. An important point to keep in mind in extending synthesis chains to first generation feedstocks of simple chemical complexity is that environmental and safety-hazard impacts of reactants and reagents become important since reaction solvent usage dramatically decreases. Reactions involving first generation feedstocks are typically gas-phase reactions run without any reaction solvent. However, as the synthesis chain extends to more complex intermediates and other materials, reaction solvent usage increases dramatically and becomes the bulk input mass of materials used. Hence, environmental and safety-hazard impacts of reaction solvents become important in the pharmaceutical industry, for example.

Deciding on where to terminate a chain is a contentious issue; however, general guidelines can be created to help direct and facilitate decision-making in the form of a decision tree. A first key question to ask is: “Does a plan trace to a renewable or reclaimed starting material?”. If the answer is “yes”, then all metrics analyses up to that renewable material need to be done. On the other hand, if the answer is “no”, then there are three possible options in decreasing order of thoroughness. The first option is to trace to a non-renewable starting material that is common to all synthesis plans compared as far as possible. The second option, if no common starting material can be found, is to trace to a non-renewable starting material (i) that is a first generation feedstock such as coal, crude oil, or ores from the earth’s crust; or (ii) whose molecular weight is less than 80 g/mol corresponding to benzene or pyridine starting materials. The third option is to trace to a “readily available” or commercially available starting material. An agreed consensus between academic, industry, and government stakeholders is needed to decide which option is most appropriate and feasible, or to decide other options. The main criterion for tracing each reaction step in a backwards fashion is to always choose literature examples with the least PMI, highest yields and atom economies, and least environmental-safety hazard impacts. As one goes towards virgin materials energy demands and hazard impacts generally increase. All synthesis plans should be compared in the same way to avoid biased ranking. For example, if reagents and catalysts are not traced further back, then this is done for all plans considered to a given target product. On the other hand, if they are traced back to earlier materials then this should be done for all plans. A key problem is that ranking positions can be arbitrarily selected for a given set of synthesis plans to a common target product simply by selecting the cut-off chain of starting materials. Hence, the computation of SI and subsequent ranking are most vulnerable to the apparent arbitrary choice of starting material cut-offs in the analyses. Transportation costs for getting starting materials to the manufacturing site also contribute to input energy; however, for the case of simplicity we have not considered these in the present analysis.

Based on the above challenges and limitations discussed above, we believe that our choice of starting materials used to carry out our metric analyses for all synthesis plans of vanillin considered as shown in Figure S1 (Supporting Information File 1) are reasonable and lead to fair ranking comparisons. We have also shown how synthesis plans may be compared and ranked using Borda count and poset pairwise dominance algorithms according to the following attributes: process mass intensity (PMI), sacrificial reagent consumption (SR), input enthalpy energy consumption (IEE), Rowan solvent greenness index (RSGI), and sustainability index (SI). The Borda count method is found to be adequate for rapid identification of best and worst plans in a given set of synthesis plans, whereas, the more detailed poset pairwise dominance analysis is appropriate for obtaining a precise ranking of intermediate performing plans. Application of these ranking methods to 22 synthesis plans for vanillin indicated that biofermentation processes from ferulic acid and isoeugenol starting materials were best performers overall followed closely by percolation solvent extraction processes from whole or cut cured vanilla beans. Chemical syntheses of vanillin from lignosulfonic acid liquors or guaiacol were surprisingly competitive when analyzed according to all of the 5 comparative attributes; however, they ranked low with respect to the sustainability index alone. The most sustainable processes were biofermentations and percolation solvent extractions.

**Table 8:** Abbreviations

Abbreviation	Explanation

ABP	acidification-basification potential
aqua	aquatic toxicity, mg/L for 96 h
BCP	bioconcentration potential, unitless
*F_VI_*	mass fraction of valorized inputs
*F_NVI_*	mass fraction of non-valorized inputs
*F_VO_*	mass fraction of valorized outputs
*F_NVO_*	mass fraction of non-valorized outputs
*F_RE_*	input enthalpic energy fraction arising from renewable energy sources
*F_VP_*	mass fraction of valorized target product
FP	flash point, degrees K
GWP	global warming potential, unitless
IEE	input enthalpic energy, kJ per kg product
*K*	number of synthesis plans to a common target product
LC_50_	lethal concentration required to kill 50% of population, g/m^3^ for 4 h
LD_50_	lethal dose required to kill 50% of population, mg/kg body weight
*m*	number of attributes used in a ranking algorithm
*M*	metric
*M_VI_*	mass of valorized or renewably sourced inputs
*M_NVI_*	mass of non-valorized or non-renewably sourced inputs
*M_product_*	mass of target product produced in a synthesis plan
M^*^_product_	mass of target product that is destined to be waste after its intended use
ODP	ozone depletion potential, unitless
OEL	occupational exposure limit, ppm
*OSI*	overall solvent index
PER	persistence potential, unitless
PMI	process mass intensity, kg total inputs per kg product
Q	quotient referring to risk phrases, unitless
*r*	rank number for a plan
RE	renewable energy
RSGI	Rowan solvent greenness index, kg per kg product
SD	skin dose, mg
SFE	supercritical fluid extraction
SFP	smog forming potential, unitless
SI	sustainability index, dimensionless
SR	sacrificial reagents
*W_VO_*	mass of waste of valorized or reusable outputs
*W_NVO_*	mass of waste of non-valorized outputs
*W_total_*	total mass of waste produced in a synthesis plan

## Supporting Information

File 1 Figure S1 comprising 22 vanillin synthesis plans, data for vanillin synthesis plans on production of 1 kg vanillin, Borda count results, and Poset analysis.

File 2 Excel file of sustainability index (SI) calculator.

File 3 Excel template file for carrying out poset pairwise dominance analysis.
