# Supplementary material for: A proposed sustainability index for synthesis plans based on input provenance and output fate: application to academic and industrial synthesis plans for vanillin as a case study
Source: Beilstein J Org Chem. 2020 Sep 25;16:2346–62. doi: 10.3762/bjoc.16.196 (PMC7522458; doi:10.3762/bjoc.16.196)
Supplement: File 1 — Figure S1 comprising 22 vanillin synthesis plans, data for vanillin synthesis plans on production of 1 kg vanillin, Borda count results, and Poset analysis. [file Beilstein_J_Org_Chem-16-2346-s001.pdf]

BEILSTEIN JOURNAL OF ORGANIC CHEMISTRY

Supporting Information

for

A proposed sustainability index for synthesis plans based on input provenance and output fate: application to academic and industrial synthesis plans for vanillin as a case study

John Andraos

Beilstein J. Org. Chem. **2020,** *16,* 2346–2362. doi:10.3762/bjoc.16.196

Figure S1 comprising 22 vanillin synthesis plans, data for vanillin synthesis plans on production of 1 kg vanillin, Borda count results, and Poset analysis

License and Terms: This is a supporting information file under the terms of the Creative Commons Attribution License (https://creativecommons.org/licenses/by/4.0). Please note that the reuse, redistribution and reproduction in particular requires that the authors and source are credited.
The license is subject to the *Beilstein Journal of Organic Chemistry* terms and conditions: (https://www.beilstein-journals.org/bjoc)

Table of Contents

Part 1: Figure S1. List of 22 vanillin synthesis plans showing reaction conditions in each reaction step, Page 3
Part 2: Data for Vanillin Synthesis Plans Based on Production of 1 kg Vanillin, Page 9
P1: Borregaard
P2: Collins Chemical
P3: Eilks – Pt 1
P4: Eilks – Pt 2
P5: Faith
P6: Frost
P7: Givaudan-Roure
P8: Haarmann & Reimer
P9: Hibbert
P10: Ji
P11: Lampman
P12: Lesage-Meesen
P13: Mayer
P14: Mexican group SFE
P15: Mottern
P16: Ontario Paper Co.
P17: Percolation extraction cut
P18: Percolation extraction whole
P19: Sorensen-Mehlum
P20: Soxhlet extraction cut
P21: Soxhlet extraction ground
P22: Taber

Part 3: Borda Count Results, Page 29

Part 4: Poset Analysis, Page 32
PMI versus SR
PMI versus IEE
PMI versus RSGI
PMI versus SI
SR versus IEE
SR versus RSGI
SR versus SI
IEE versus RSGI
IEE versus SI
RSGI versus SI

Part 1: Figure S1. List of 22 vanillin synthesis plans showing reaction conditions in each reaction step.

p1: Borregaard (1999)

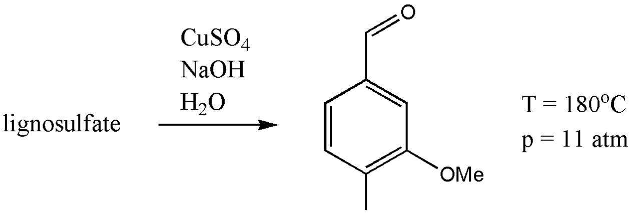

p2: Collins Chemical (1970)

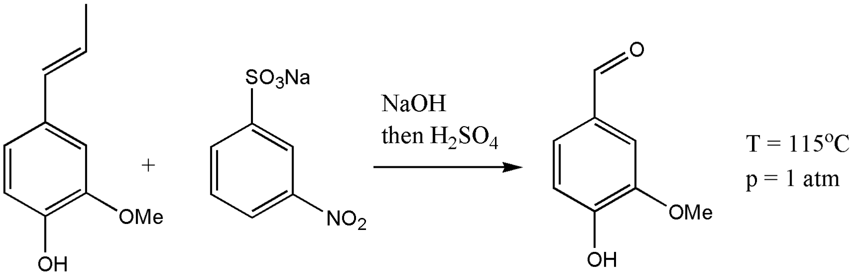

p3: Eilks - Pt 1 (2016)

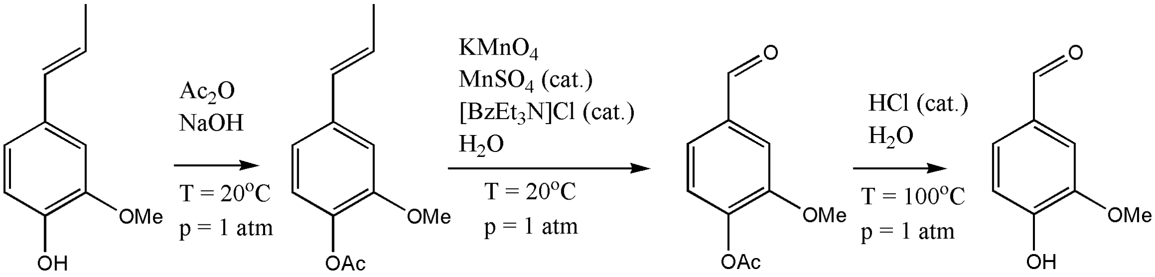

p4: Eilks - Pt 2 (2016)

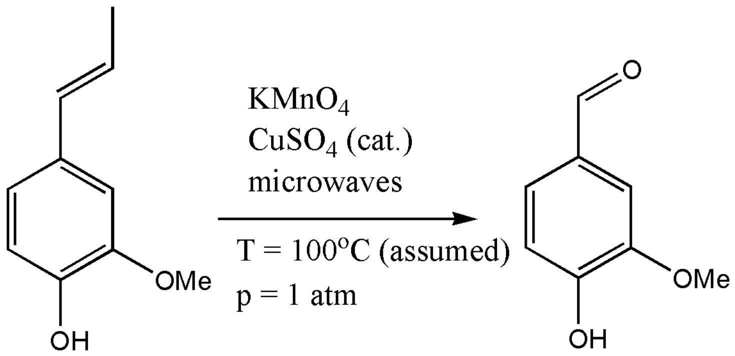

p5: Faith (1966)

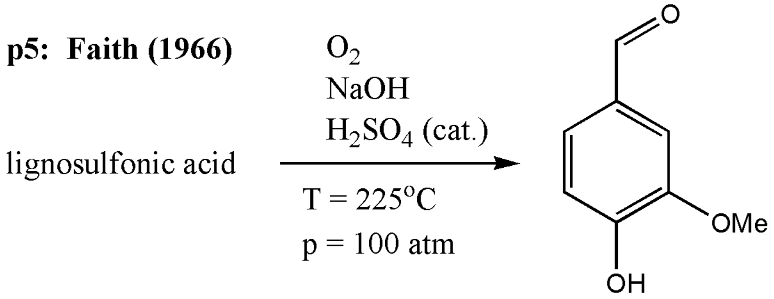

p6: Frost (1998)

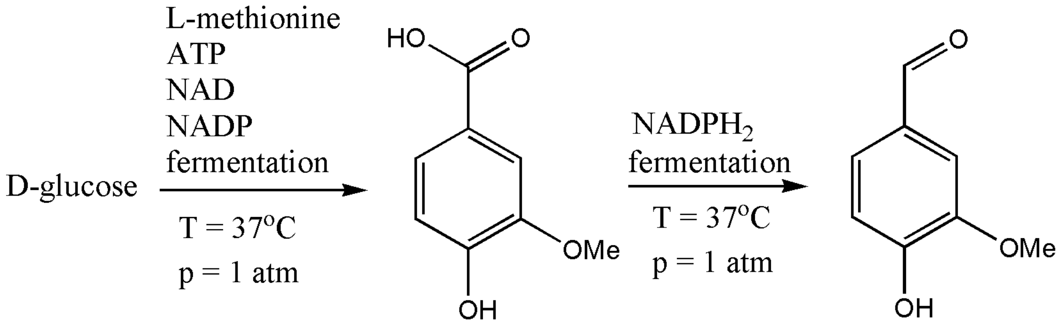

p7: Givaudan-Roure (1998)

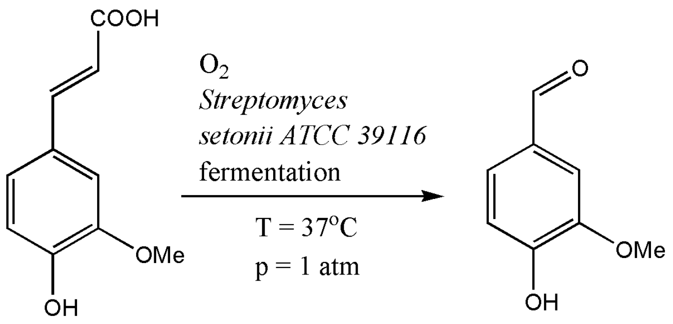

p8: Haarmann-Reimer (1991)

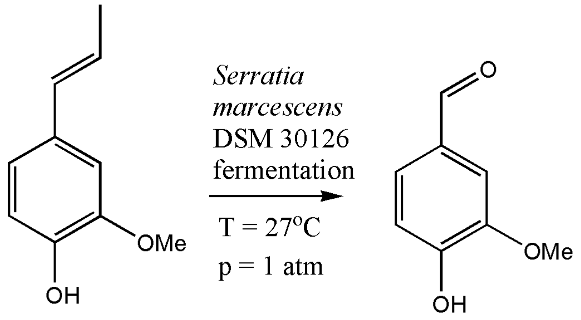

p9: Hibbert (1936)

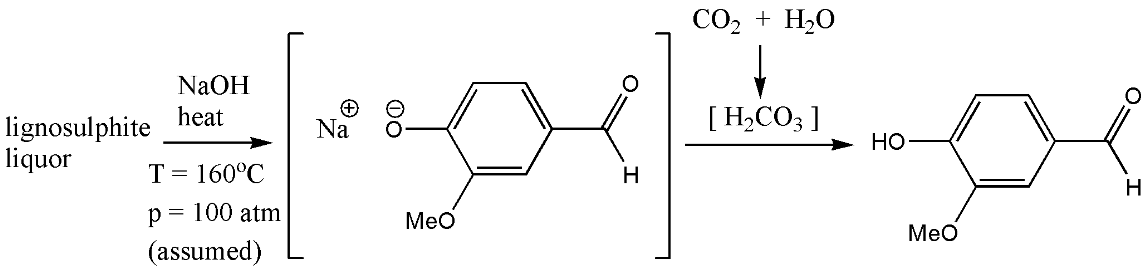

p10: Ji (2013)

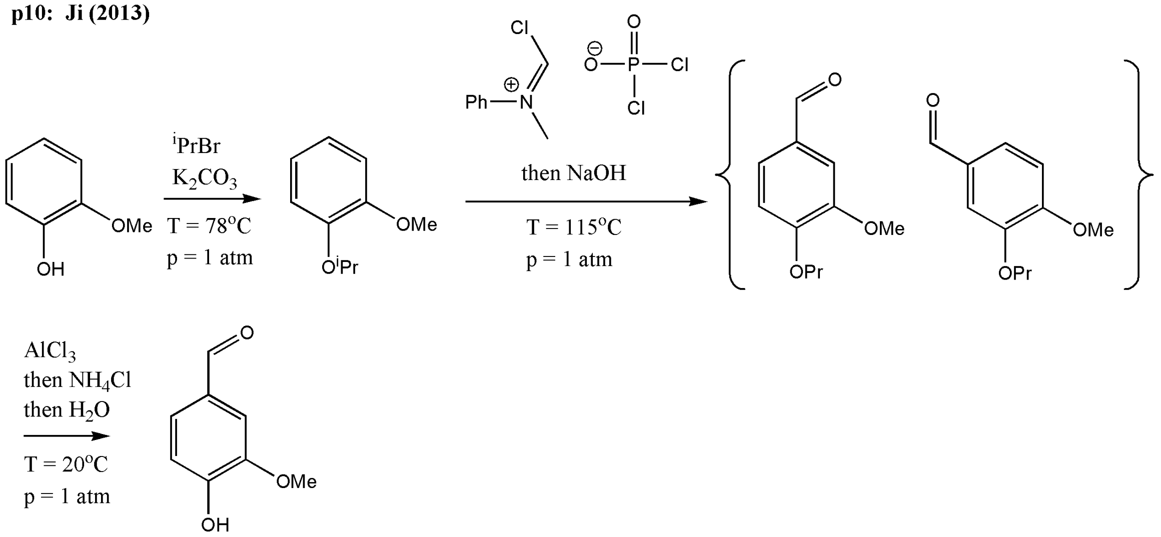

p11: Lampman (1977)

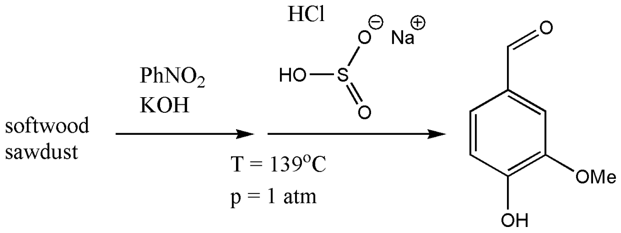

p12: Lesage-Meesen (1996)

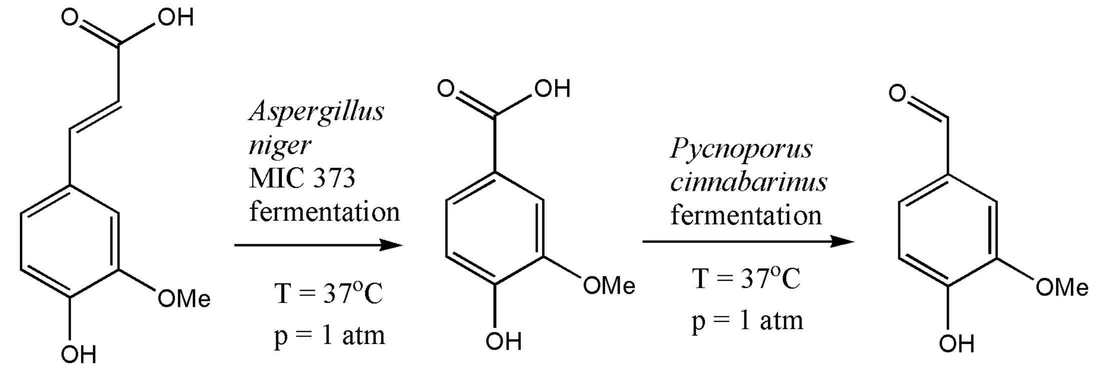

p13: Mayer (1949)

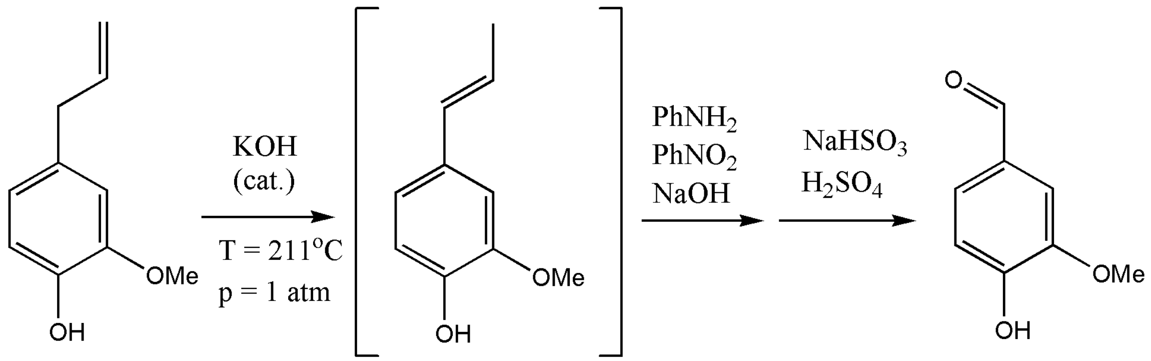

p14: Mexican group SFE (2011)

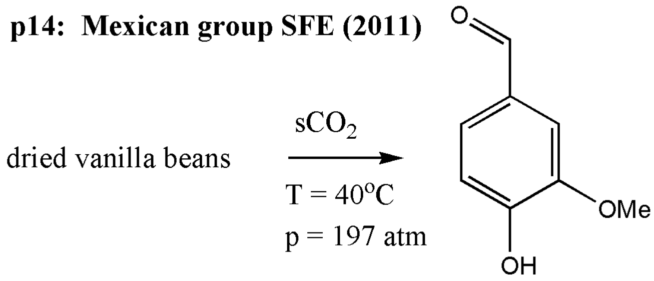

p15: Mottern (1934)

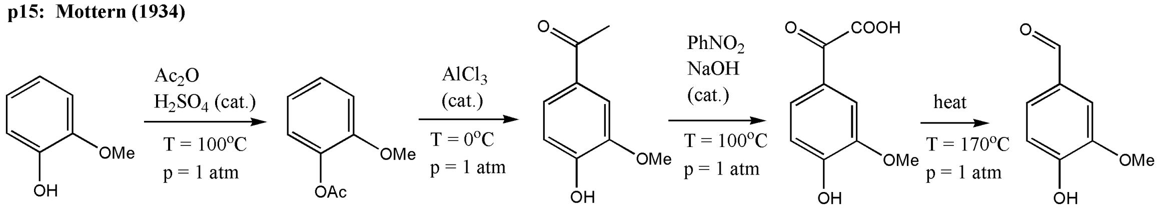

p16: Ontario Paper Co. (1962)

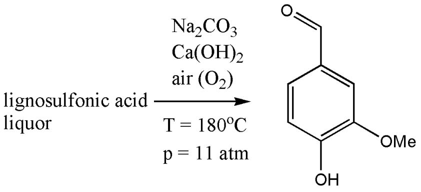

p17: percolation extraction cut (2005)

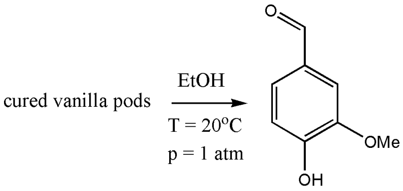

p18: percolation extraction whole (2005)

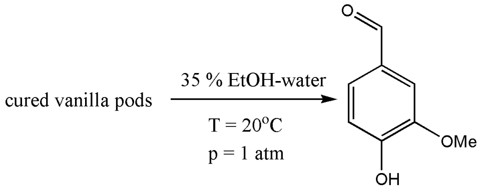

p19: Sorensen-Mehlum (1956)

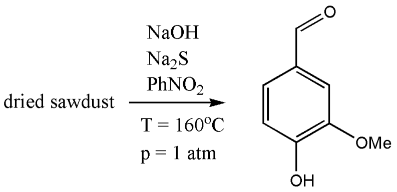

p20: Soxhlet extraction cut (1995)

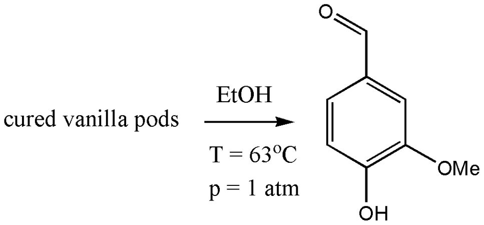

p21: Soxhlet extraction ground (1995)

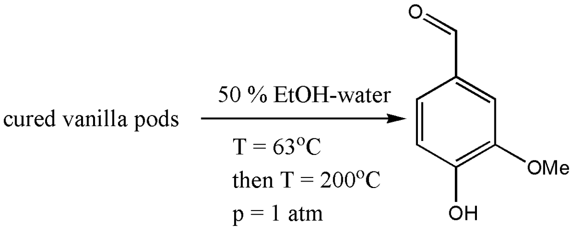

p22: Taber (2007)

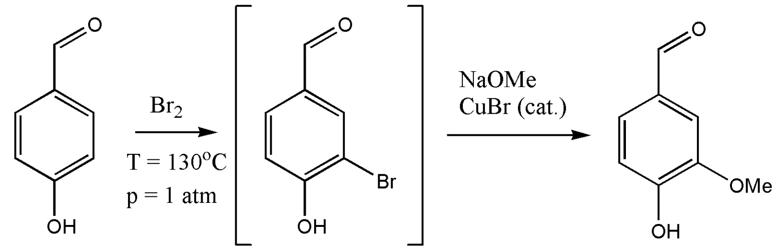

Part 2: Data for Vanillin Synthesis Plans Based on Production of 1 kg Vanillin

P1: Borregaard

Bjørsvik, H.R. Org. Process Res. Dev. 1999, 3, 330-340. DOI:10.1021/op9900028

**Input Materials**	
**Reagents**	
lignosulfate (24 wt%)	12500g
CuSO4 (16.3 wt%)	182.14g
NaOH (50 wt%)	8377.59g
**Reaction Solvents**	
water from lignosulfate	39583.33g
water from CuSO4 soln.	935.28g
water from NaOH soln.	8377.59g
water	14015.15g
**Catalysts**	
none	0g
**Workup**	
none	0g
**Purification**	
none	0g
**SUM**	83971.10g
**Waste Byproducts**	
unidentified	20059.73g
**sacrificial reagents**	
CuSO4 (16.3 wt%)	182.14g
NaOH (50 wt%)	8377.59g
	8559.73g

P2: Collins Chemical

Fiecchi, A.; Mario, G.; Cabella, P.; Cicognani, G. US3544621 (1970)

**Input Materials**	
**Reagents**	
isoeugenol	1199.04g
NaOH	1199.04g
m-NO2-benzenesulfonate Na	2398.08g
H2SO4 (96 wt%)	4834.53g
**Reaction Solvents**	
water	19184.65g
benzene	3161.87g
water from 96 wt% H2SO4	201.44g
**Catalysts**	
65 wt% HNO3	1601.50g
**Workup Materials**	
NaOH	1973.28g
water	1973.28g
**Purification**	

none	0g
**SUM**	37726.71g

Waste Byproducts

water	118.42g
acetaldehyde	289.47g
azobenzene byproduct	1269.74g
NaHSO4	789.47g
unidentified	6163.59g
	8630.70g

sacrificial reagents

NaOH	1199.04g

P3: Eilks – Pt 1

Garner, N.; Siole, A.; Eilks, I. J. Sci. Educ. 2016, 17, 25-28.

Step 1

Input Materials

**Reagents**	
isoeugenol	8902.05g
acetic anhydride	14891.86g
NaOH (1 M, 3.8 wt%)	10815.26g
**Reaction Solvents**	
water from 1 M NaOH	273796.94g
**Catalysts**	
none	0g
**Workup**	
none	
**Purification**	
EtOH	54346.33g
water	68880.01g
**SUM**	431632.45g

Waste Byproducts

water	734.27g
NaOAc	3345.03g
unidentified	22126.51g
	26205.81g

sacrificial reagents

acetic anhydride	14891.86g
NaOH (1 M, 3.8 wt%)	10815.26g
	25707.12g

Step 2

Input Materials

Reagents

product 2	8403.36g
KMnO4	15966.39g
water	315126.05g
**Reaction Solvents**	
MTBE	233193.28g
**Catalysts**	
MnSO4	15966.39g
[BzEt3N]Cl	840.34g
**Workup Materials**	
MTBE	248739.49g
NaHCO3	4201.68g
water	84033.61g
**Purification**	
none	0g
**SUM**	918067.22g

**Waste Byproducts**	
MnO2	1908.07g
KOH	1229.03g
acetaldehyde	724.25g
unidentified	332441.18g
	336302.52g

**sacrificial reagents**	
water	315126.05g

Step 3

**Input Materials**	
**Reagents**	
product 2	3193.28g
water (as 6 M HCl, 20 wt%)	110823.53g
**Reaction Solvents**	
none	0g
**Catalysts**	
HCl	27705.88g
**Workup Materials**	
MTBE	93277.31g
activated charcoal	4201.68g
**Purification**	
water	126050.42g
**SUM**	362058.82g

**Waste Byproducts**	
HOAc	394.74g
unidentified	112622.07g
	113016.81g

**sacrificial reagents**	
none	0g

Overall

	mass inputs (g)
step 1	431632.5
step 2	918067.2
step 3	362058.8
SUM	1711758

product		
vanillin	1000	g

sacrificial reagents	mass (g)
step 1	25707.12
step 2	315126.1
step 3	0
SUM	340833.2

P4: Eilks – Pt 2

Garner, N.; Siole, A.; Eilks, I. J. Sci. Educ. 2016, 17, 25-28.

**Input Materials**	
**Reagents**	
isoeugenol	1687.24g
KMnO4	10823.05g
**Reaction Solvents**	
none	0.00g
**Catalysts**	
CuSO4	43621.40g
**Workup Materials**	
MTBE	91358.02g
**Purification**	
none	0g
**SUM**	147489.71g
**Waste Byproducts**	
KMnO2	649.18g
acetaldehyde	226.80g
unidentified	10634.31g
	11510.29g
**sacrificial reagents**	
none	0.00g

P5: Faith

Faith, W.L.; Keyes, D.B.; Clark, R.L. Industrial Chemicals, 3rd ed., Wiley: New York, 1966, p. 796

**Input Materials**	
**Reagents**	
lignosulfonic acid	6700g
dioxygen	5147.05g
NaOH	20000g
**Reaction Solvents**	
butanol	1000g
nitrogen	16727.91g
**Catalysts**	
H2SO4 (66 Be)	400g
**Workup**	
none	0g
**Purification**	
none	0g
**SUM**	49974.96g

**Waste Byproducts**	
unidentified	30847.05g

**sacrificial reagents**	
none	0g

P6: Frost

Li, K.; Frost, J.W. J. Am. Chem. Soc. 1998, 120, 10545

Step 1

**Input Materials**	
**Reagents**	
L-methionine	2222.22g
D-glucose	111111.11g
ATP	22777.78g
NAD	11111.11g
NADP	45555.56g
**Reaction Solvents**	
water	11111111.33g
**Catalysts**	
nutrients	383888.90g
**Workup**	
none	0g
**Purification**	
none	0g
**SUM**	11687778.01g

Waste byproducts

ADP	12708.33g
NADH2	6736.11g
NADPH2	30119.05g
CO2	873.02g
fructose 6-phosphate	2579.37g
H3PO4	972.22g
2-amino-4-mercapto-butyric acid	1339.29g
dihydroxyacetone phosphate	1686.51g
unidentified	134097.22g
**SUM**	191111.11g

sacrificial reagents

ATP	22777.78g
NAD	11111.11g
NADP	45555.56g
**SUM**	79444.45g

Step 2

Input Materials

**Reagents**	
vanillic acid	1666.67g
NADPH2	12666.67g
**Reaction Solvents**	
fermentation broth	366666.67g
**Catalysts**	
nutrients	295233.33g
**Workup**	
EtOAc	901000.00g
water	1373333.33g
CH2Cl2	1336000.00g
**Purification**	
none	0g
SUM	4284900.00g

Waste byproducts

NADP	4980.26g
water	118.42g
unidentified	8234.65g
**SUM**	13333.33g

sacrificial reagents

NADPH2	12666.67g

Overall

Input materials

step 1	11687778g
step 2	4284900g

SUM	15972678	g

Product

vanillin	1000	g

sacrificial reagents

step 1	79444.45	
step 2	12666.67	
SUM	92111.12	g

P7: Givaudan-Roure
Muheim, A.; Müller, B.; Münch, T.; Wetli, M. EP885968 (Givaudan-Roure, 1998)

**Input Materials**	
**Reagents**	
ferulic acid (10 wt% in 0.5 M NaOH)	1700g
dioxygen	740.74g
**Reaction Solvents**	
0.5 M NaOH soln	15300.00g
**Catalysts**	
nutrients	3038.52g
**Workup**	
none	0g
**Purification**	
none	0g
**SUM**	20779.26g

**Waste Byproducts**	
glyoxylic acid	486.84g
unidentified	953.90g

**sacrificial reagents**	
none	0g

P8: Haarmann & Reimer
Rabenhorst, J.; Hopp, R. US5017388 (1991)

**Input Materials**	
**Reagents**	
isoeugenol	22222.22g
**Reaction Solvents**	
water	1111111.11g
**Catalysts/Nutrients**	
Na2HPO4	3472.22g
KH2PO4	2777.78g
(NH4)2PO4	2777.78g
FeSO4 7 H2O	27.78g
MgSO4 (1 M)	2222.22g
CaCl2 (0.1 M)	3333.33g

glycerol	11111.11g
**Workup**	
none	0g
**Purification**	
none	0g
**SUM**	1159055.56g

**Waste Byproducts**	
acetaldehyde	289.47g
unidentified	20932.75g
	21222.22g

**sacrificial reagents**	
none	0g

P9: Hibbert

Hibbert, H.; Tomlinson, G.H. Jr. US 2069185 (1937)

**Input Materials**	
**Reagents**	
sulphite liquor	375000g
NaOH	42857.14g
CO2	71428.57g
water	0.00g
**Reaction Solvents**	
none	0g
**Catalysts**	
none	0g
**Workup Materials**	
benzene	313928.57g
**Purification Materials**	
none	0g
**SUM**	803214.29g

**Waste Byproducts**	
unidentified	488285.71g

**sacrificial reagents**	
CO2	71428.57g

P10: Ji

Huang, W.B.; Du, C.Y.; Jiang, J.A.; Ji, Y.F. Res. Chem. Intermed. 2013, 39, 2849-2856. DOI:10.1007/s11164-012-0804-6

Step 1

Input Materials

Reagents

guaiacol	6877.38g
iPrBr	13632.74g
K2CO3	15307.72g
**Reaction Solvents**	
EtOH	67355.56g
**Catalysts**	
KI	1885.73g
**Workup**	
none	
**Purification**	
silica gel	0.00g
CH2Cl2	1425649.93g
**SUM**	1530709.07g

**Waste Byproducts**	
KBr	5800.00g
KHCO3	4878.05g
unidentified	17042.23g
	27720.28g

**sacrificial reagents**	
iPrBr	13632.74g
K2CO3	15307.72g
	28940.46g

Step 2

**Input Materials**	
**Reagents**	
product 2	8097.56g
O=PCl3	11219.51g
PhNHCHO	9878.05g
NaOH (2 M)	9756.10g
**Reaction Solvents**	
water from 2 M NaOH	121951.22g
**Catalysts**	
none	0.00g
**Workup Materials**	
CH2Cl2	733170.73g
**Purification**	
none	0g
**SUM**	885975.61g

**Waste Byproducts**	
water	735.48g
NaCl	2388.26g
PhNHMe	4372.01g
Na[O=PCl2O]	6410.93g
unidentified	17117.71g
	31024.39g

sacrificial reagents

O=PCl3	11219.51g
NaOH	4939.02g
	16158.54g

Step 3

**Input Materials**	
**Reagents**	
product 2	7926.83g
water as NH4Cl soln.	60975.61g
**Reaction Solvents**	
CH2Cl2	48878.05g
**Catalysts**	
AlCl3	2927.20g
NH4Cl (2 M)	6396.34g
**Workup Materials**	
CH2Cl2	244390.24g
sat'd NaCl soln.	172741.46g
**Purification**	
silica gel	0.00g
pet.ether	780487.80g
EtOAc	109878.05g
**SUM**	1426674.76g

Waste Byproducts

iPrOH	394.74g
unidentified	67507.70g
	67902.44g

sacrificial reagents

none	0g

Overall

	mass inputs (g)
step 1	1530709
step 2	885975.6
step 3	1426675
SUM	3843359

product	
vanillin	1000g

sacrificial reagents	mass (g)
step 1	28940.46
step 2	16158.54
step 3	0
SUM	45099g

P11: Lampman

Lampman, G.M.; Andrews, J.; Bratz, W.; Hanssen, O.; Kelley, K.; Perry, D.; Ridgeway, A. J. Chem. Educ. 1977, 54, 776

**Input Materials**	
**Reagents**	
sawdust	457317.07g
PhNO2	1467073.17g
KOH	579268.29g
HCl (37 wt%)	1948774.39g
NaHSO3 (20 wt%)	7317073.17g
**Reaction Solvents**	
DMSO	2685365.85g
water	762195.12g
**Catalysts**	
none	0g
**Workup Materials**	
water	6097560.98g
Et2O	9713414.63g
MgSO4	609756.10g
**Purification Materials**	
hot cyclohexane	593750.00g
cyclohexane	308750.00g
SUM	32540298.78g

**Waste Byproducts**	
unidentified	11768506.10g

**sacrificial reagents**	
KOH	579268.29g
HCl (37 wt%)	1948774.39g
NaHSO3 (20 wt%)	7317073.17g
SUM	9845115.85g

P12: Lesage-Meesen

Lesage-Meesen, L.; Delattrea, M.; Haona, M.; Asther, M. US 6162637 (Institut National de la Recherche Agronomique, 2000)

Lesage-Meesen, L.; Delattrea, M.; Haona, M.; Thibault, J.F.; Ceccaldi, B.C.; Brunerie, P.; Asther, M. J. Biotechnology 1996, 50, 107

Step 1

**Input Materials**	
**Reagents**	
ferulic acid	9698.28g
dioxygen	4105.09g
**Reaction Solvents**	
water	1436781.61g
**Catalysts**	

nutrients	57060.76g
**Workup**	
none	0g
**Purification**	
none	0g
**SUM**	1507645.73g

**Waste Byproducts**	
oxalic acid	2770.94g
unidentified	5860.02g
	8630.95g

**sacrificial reagents**	
none	0g

Step 2

**Input Materials**	
**Reagents**	
vanillic acid	5172.41g
NADPH2	29556.65g
**Reaction Solvents**	
water	17241379.31g
**Catalysts**	
nutrients	684729.06g
**Workup**	
none	0g
**Purification**	
none	0g
**SUM**	17955665.02g

**Waste Byproducts**	
water	118.42g
NADP	4980.26g
unidentified	28630.38g
	33729.06g

**sacrificial reagents**	
NADPH2	29556.65g

Overall

Input materials	
step 1	1507646g
step 2	17926207g
SUM	19433853g

product	
vanillin	1000g

**sacrificial reagents**	
step 1	0

step 2	29556.65	
SUM	29556.65	g

P13: Mayer

Mayer, E. Oesterreichische Chem. Ztg. 1949, 50, 40

**Input Materials**	
**Reagents**	
eugenol	1171.43g
PhNH2	5986.00g
PhNO2	4210.50g
NaOH (50 wt%)	1035.03g
H2SO4 (50 wt%)	1993.00g
NaHSO3 (20 wt%)	857.14g
**Reaction Solvents**	
water	642.86g
water from 50 wt% NaOH	1035.03g
water from 20 wt% NaHSO3	3428.57g
**Catalysts**	
KOH	607.14g
**Workup**	
benzene	4897.29g
**Purification**	
none	0g
**SUM**	25863.98g

**Waste Byproducts**	
water	473.68g
PhN=NPh	2394.74g
CH3CHO	289.47g
NaHSO4	1578.95g
SO2	421.05g
unidentified	9095.20g
	14253.10g

**sacrificial reagents**	
PhNH2	5986.00g
NaOH (50 wt%)	1035.03g
H2SO4 (50 wt%)	1993.00g
NaHSO3 (20 wt%)	857.14g
**SUM**	9871.17g

P14: Mexican group SFE

Castillo-Ruz, M.C.; Guillermo-Alcocer, C.G.; Bojorquez-Gamboa, R.R.; Rocha-Uribe, J.A. Tecnol. Ciencia Ed. (IMIQ) 2011, 26, 80-84.

Input Materials

Reagents

dried vanilla beans	17241.38g
**Reaction Solvents**	
sCO2	7496251.87g
**Catalysts**	
none	0g
**Workup**	
none	0g
**Purification**	
none	0g
**SUM**	7513493.25g
**sacrificial reagents**	
none	0g
**Waste Byproducts**	
spent vanilla beans	16241.38g

P15: Mottern

Mottern, H.O. J. Am. Chem. Soc. 1934, 56, 2107-2108.

Step 1

**Input Materials**	
**Reagents**	
guaiacol	2046.26g
acetic anhydride	1848.23g
**Reaction Solvents**	
none	0g
**Catalysts**	
H2SO4 (98 wt%)	30.30g
**Workup Materials**	
water	33004.12g
Na2CO3	3300.41g
**Purification**	
none	0g
SUM	40229.32g
**Waste Byproducts**	
HOAc	966.27g
unidentified	254.89g
	1221.15g
**sacrificial reagents**	
none	0g

Step 2

**Input Materials**	
**Reagents**	
product 1	2673.33g
**Reaction Solvents**	
none	0g

**Catalysts**	
AlCl3	2744.62g
**Workup Materials**	
ice	35644.45g
Et2O	25236.27g
Na2SO4	3564.45g
**Purification**	
none	0g
SUM	67189.79g
**Waste Byproducts**	
unidentified	1318.84g
**sacrificial reagents**	
none	0g

Step 3

**Input Materials**	
**Reagents**	
product 2	1354.49g
PhNO2	1044.89g
**Reaction Solvents**	
water	2902.48g
**Catalysts**	
NaOH	967.49g
**Workup Materials**	
HCl	967.49g
charcoal	967.49g
**Purification**	
none	0g
SUM	6849.85g
**Waste Byproducts**	
aniline	624.33g
unidentified	459.26g
	1083.59g
**sacrificial reagents**	
none	0g

Step 4

**Input Materials**	
**Reagents**	
product 3	1315.79g
**Reaction Solvents**	
p-Me2N-C6H4-CH3	1315.79g
**Catalysts**	
none	0g
**Workup Materials**	

Et2O	3726.32g
dilute HCl	5368.42g
**Purification**	
none	0g
SUM	10410.53g

Waste Byproducts

CO2	289.47g
unidentified	26.32g
	315.79g

sacrificial reagents

none	0g

Overall

Input materials

step 1	40229.32g
step 2	67189.79g
step 3	6849.85g
step 4	10410.53g
SUM	124679.5g

product

vanillin	1000g

sacrificial reagents

step 1	0g
step 2	0g
step 3	0g
step 4	0g
SUM	0g

P16: Ontario Paper Co.
Craig, D.; Logan, C.D. US3054659 (1962)

Input Materials

**Reagents**	
lignosulfonic acid liquor	245542.23g
Na2CO3	18833.33g
Ca(OH)2	13666.67g
air (oxygen)	49600.43g
**Reaction Solvents**	
nitrogen	161201.41g
**Catalysts**	
none	0g
**Workup**	
H2SO4	35491.50g
toluene	206830.32g
**Purification**	

none	0g
**SUM**	731165.90g

**Waste Byproducts**	
solids	26333.19g
lignin	5249.97g
acetovanillone	83.33g
calcium oxalate	500.00g
sodium sulfate	25166.53g
unidentified	269309.64g
	326642.66g

**sacrificial reagents**	
Na2CO3	18833.33g
Ca(OH)2	13666.67g
	32500.00g

P17: Percolation extraction cut
Sujalmi, S.; Suharso; Supriyanto, R.; Buchari Indo. J. Chem. 2005, 5, 7-10.

**Input Materials**	
**Reagents**	
cured vanilla pods	49504.95g
**Reaction Solvents**	
none	0g
**Catalysts**	
none	0g
**Workup**	
EtOH	781188.12g
**Purification Materials**	
none	0g
**SUM**	830693.07g

**Waste Byproducts**	
spent vanilla pods	48504.95g

**sacrificial reagents**	
none	0g

P18: Percolation extraction whole
Sujalmi, S.; Suharso; Supriyanto, R.; Buchari Indo. J. Chem. 2005, 5, 7-10.

**Input Materials**	
**Reagents**	
vanilla pods	83252.57g
**Reaction Solvents**	
35 wt% EtOH-water	944932.33g
**Catalysts**	
none	0g

**Workup**	
none	0g
**Purification**	
none	0g
**SUM**	1028184.90g

**Waste Byproducts**	
spent vanilla pods	82253g

**sacrificial reagents**	
none	0g

P19: Sorensen-Mehlum
Sorensen, N.A.; Mehlum, J. US2752394 (1956)

**Input Materials**	
**Reagents**	
dried sawdust	11382.11g
NaOH	22926.83g
Na2S	6585.37g
PhNO2	48902.44g
**Reaction Solvents**	
water	243902.44g
**Catalysts**	
none	0g
**Workup**	
H2SO4	56170.73g
Et2O	172682.93g
**Purification**	
none	0g
**SUM**	562552.85g

**Waste Byproducts**	
unidentified	88796.75g

**sacrificial reagents**	
NaOH	22926.83g
Na2S	6585.37g
	29512.20g

P20: Soxhlet extraction cut
Sujalmi, S.; Suharso; Supriyanto, R.; Buchari Indo. J. Chem. 2005, 5, 7-10.

**Input Materials**	
**Reagents**	
cured vanilla pods	28089.89g
**Reaction Solvents**	
none	0g
**Catalysts**	

none	0g
**Workup**	
EtOH	554073.03g
**Purification Materials**	
none	0g
**SUM**	582162.92g
**Waste Byproducts**	
spent vanilla pods	27089.89g
**sacrificial reagents**	
none	0g

P21: Soxhlet extraction ground
Voisine, R.; Carmichael, L.; Chalier, P.; Cormier, F.; Morin, A. J. Agr. Food Chem. 1995, 43, 2658-2661. DOI:10.1021/jf00058a019

**Input Materials**	
**Reagents**	
cured vanilla pods	56000g
**Reaction Solvents**	
none	0g
**Catalysts**	
none	0g
**Workup**	
50 % (v/v) EtOH	3924480g
Et2O	708000g
95 % (v/v) EtOH	204025g
silica gel	17500g
water	250000g
methanol	59400g
47.5 % (v/v) EtOH	18783.2g
**Purification**	
47.5 % (v/v) EtOH	93916g
**SUM**	5332104.2g
**Waste Byproducts**	
spent vanilla pods	55000g
**sacrificial reagents**	
none	0g

P22: Taber
Taber, D.F.; Patel, S.; Hambleton, T.M.; Winkel, E.E. J. Chem. Educ. 2007, 84, 1158. DOI:10.1021/ed084p1158

**Input Materials**	
**Reagents**	
4-hydroxybenzaldehyde	1084.60g

bromine	1420.65g
NaOMe	480.07g
**Reaction Solvents**	
methanol	25770.07g
EtOAc	977.22g
**Catalysts**	
CuBr	867.68g
**Workup Materials**	
3 M HCl soln.	68329.72g
EtOAc	146583.51g
Na2SO4	10845.99g
**Purification Materials**	
silica gel	59652.93g
Et2O	230368.76g
pet. Ether	1180043.38g
water	216919.74g
**SUM**	1943344.32g
**Waste Byproducts**	
HBr	532.24g
NaBr	676.97g
unidentified	776.10g
	1985.31g
**sacrificial reagents**	
bromine	1420.65g

Part 3: Borda Count Results

Plan	PMI (kg/kg)	Points
Givaudan-Roure	21	22
Mayer	26	21
Faith	50	20
Collins Chemical	38	19
Borregaard Synthesis	84	18
Mottern	125	17
Eilks Pt 2	147	16
Sorensen-Mehlum	563	15
Soxhlet extraction cut	582	14
Ontario Paper Co.	730	13
Hibbert	803	12
percolation extraction cut	831	11
percolation extraction whole	1028	10
Haarmann & Reimer	1159	9
Eilks Pt 1	1712	8
Taber	1943	7
Ji	3843	6
Soxhlet extraction ground	5332	5
Mexican group SFE	7513	4
Frost	15973	3
Lesage-Meesen	19434	2
Lampman	32540	1

Plan	SR (kg/kg)	Points
Faith	0	22
Givaudan-Roure	0	22
Mottern	0	22
Eilks Pt 2	0	22
Haarmann & Reimer	0	22
percolation extraction cut	0	22
percolation extraction whole	0	22
Soxhlet extraction cut	0	22
Soxhlet extraction ground	0	22
Mexican group SFE	0	22
Collins Chemical	1.2	21
Taber	1.4	20
Borregaard Synthesis	8.6	19
Mayer	10	18
Sorensen-Mehlum	29.5	17
Lesage-Meesen	30	16
Ontario Paper Co.	32.5	15
Ji	45	14
Hibbert	71	13
Frost	92	12
Eilks Pt 1	341	11

Lampman	9845	10

Plan	IEE (kJ/kg)	Points
percolation extraction cut	0	22
percolation extraction whole	0	22
Givaudan-Roure	795	21
Mottern	2189	20
Eilks Pt 2	2881	19
Haarmann & Reimer	9388	18
Eilks Pt 1	17309	17
Mayer	20989	16
Faith	29286	15
Taber	28138	14
Soxhlet extraction cut	50219	13
Collins Chemical	50440	12
Borregaard Synthesis	169046	11
Ji	325200	10
Frost	578763	9
Sorensen-Mehlum	653838	8
Ontario Paper Co.	688521	7
Soxhlet extraction ground	676694	6
Lesage-Meesen	939475	5
Hibbert	1005329	4
Mexican group SFE	1678005	3
Lampman	2398980	2

Plan	RSGI (kg)	Points
Givaudan-Roure	0	22
Lesage-Meesen	0	22
Borregaard Synthesis	0	22
Haarmann & Reimer	0	22
Faith	6	21
Collins Chemical	38	20
Mayer	163	19
Mottern	277	18
Eilks Pt 2	673	17
percolation extraction whole	1579	16
Sorensen-Mehlum	1952	15
Ontario Paper Co.	2101	14
Soxhlet extraction cut	2644	13
percolation extraction cut	3727	12
Hibbert	3768	11
Eilks Pt 1	4492	10
Taber	12327	9
Frost	16369	8
Soxhlet extraction ground	16828	7
Ji	24670	6
Mexican group SFE	27578	5

Lampman	112106	4

Plan	SI	Points
Lesage-Meesen	0.8072	22
Haarmann & Reimer	0.7762	21
percolation extraction whole	0.7272	20
Frost	0.6725	19
percolation extraction cut	0.6391	18
Soxhlet extraction cut	0.6385	17
Mexican group SFE	0.6345	16
Givaudan-Roure	0.5956	15
Borregaard Synthesis	0.5756	14
Eilks Pt 1	0.4866	13
Ontario Paper Co.	0.4689	12
Mottern	0.4603	11
Faith	0.4551	10
Collins Chemical	0.4519	9
Soxhlet extraction ground	0.4246	8
Hibbert	0.4207	7
Sorensen-Mehlum	0.4171	6
Taber	0.3946	5
Ji	0.3914	4
Eilks Pt 2	0.3907	3
Lampman	0.3704	2
Mayer	0.3676	1

Part 4: Poset Analysis

Green-coloured entries indicate comparable pairs. Dominant plan is specified.
Red-coloured entries indicate incomparable pairs.
For each attribute order, plans enclosed in boxes have the same high rank.

PMI versus SR

PMI order	SR order
p7	p4
p13	p5
p2	p7
p5	p8
p1	p14
p15	p15
p4	p17
p19	p18
p20	p20
p16	p21
p9	p2
p17	p22
p18	p1
p8	p13
p3	p19
p22	p12
p10	p16
p21	p10
p14	p9
p6	p6
p12	p3
p11	p11

	p1	p2	p3	p4	p5	p6	p7	p8	p9	p10	p11	p12	p13	p14	p15	p16	p17	p18	p19	p20	p21	p22
p1		p2	p1		p5	p1	p7		p1	p1	p1	p1				p1			p1			
p2			p2			p2	p7		p2	p2	p2	p2				p2			p2			p2
p3				p4	p5		p7	p8	p9		p3		p13		p15	p16	p17	p18	p19	p20		
p4						p4			p4	p4	p4	p4				p4			p4			p4
p5						p5			p5	p5	p5	p5				p5			p5			p5
p6							p7	p8	p9	p10	p6		p13	p14	p15	p16	p17	p18	p19	p20	p21	p22
p7									p7	p7	p7	p7	p7			p7			p7			p7
p8										p8	p8	p8										p8
p9											p9		p13		p15	p16			p19	p20		
p10											p10		p13		p15	p16	p17	p18	p19	p20		p22
p11												p12	p13	p14	p15	p16	p17	p18	p19	p20	p21	p22
p12													p13	p14	p15		p17	p18	p19	p20	p21	p22
p13																p13			p13			
p14																						
p15																p15			p15			p15
p16																			p19	p20		
p17																						p17
p18																						p18
p19																						
p20																						p20
p21																						
p22																						

	p1	p2	p3	p4	p5	p6	p7	p8	p9	p10	p11	p12	p13	p14	p15	p16	p17	p18	p19	p20	p21	p22
PMI vs SR	8	10	1	9	10	1	12	6	3	2	0	1	8	3	9	5	6	6	7	8	3	4

PMI versus IEE

PMI order	IEE order
p7	p17
p13	p18
p2	p7
p5	p15
p1	p4
p15	p8
p4	p3
p19	p13
p20	p22
p16	p5
p9	p20
p17	p2
p18	p1
p8	p10
p3	p6
p22	p19
p10	p21
p21	p16
p14	p12

p6	p9
p12	p14
p11	p11

	p1	p2	p3	p4	p5	p6	p7	p8	p9	p10	p11	p12	p13	p14	p15	p16	p17	p18	p19	p20	p21	p22
p1		p2			p5	p1	p7		p1	p1	p1	p1	p13	p1		p1			p1		p1	
p2						p2	p7		p2	p2	p2	p2	p13	p2		p2			p2		p2	
p3				p4		p3	p7	p8		p3	p3	p3		p3	p15		p17	p18			p3	p3
p4						p4	p7	p4	p4	p4	p4	p4		p4	p15	p4			p4	p4	p4	p4
p5						p5	p7		p5	p5	p5	p5	p13	p5		p5			p5	p5	p5	
p6							p7	p8	p9	p10	p6	p6	p13		p15		p17	p18		p20		p22
p7								p7	p7	p7	p7	p7	p7	p7	p7	p7			p7	p7	p7	p7
p8										p8	p8	p8		p8	p15		p17	p18			p8	p8
p9											p9		p13	p9	p15	p16			p19	p20		
p10											p10	p10	p13	p10	p15		p17	p18		p20	p10	p22
p11												p12	p13	p14	p15	p16	p17	p18	p19	p20	p21	p22
p12													p13		p15	p16	p17	p18	p19	p20	p21	p22
p13														p13		p13			p13	p13	p13	p13
p14															p15	p16	p17	p18	p19	p20	p21	p22
p15																p15			p15	p15	p15	p15
p16																			p19	p20		
p17																					p17	p17
p18																					p18	p18
p19																					p19	
p20																					p20	
p21																						p22
p22																						

	p1	p2	p3	p4	p5	p6	p7	p8	p9	p10	p11	p12	p13	p14	p15	p16	p17	p18	p19	p20	p21	p22
PMI vs IEE	9	10	7	13	11	8	19	2	3	5	0	1	14	1	9	4	9	9	6	8	3	6

PMI versus RSGI

PMI order	RSGI order
p7	p1
p13	p7
p2	p8
p5	p12
p1	p5
p15	p2
p4	p13
p19	p15
p20	p4
p16	p18
p9	p19
p17	p16

p18	p20
p8	p17
p3	p9
p22	p3
p10	p22
p21	p6
p14	p21
p6	p10
p12	p14
p11	p11

	p1	p2	p3	p4	p5	p6	p7	p8	p9	p10	p11	p12	p13	p14	p15	p16	p17	p18	p19	p20	p21	p22
p1			p1	p1		p1	p7	p1	p1	p1	p1	p1		p1	p1	p1	p1	p1	p1	p1	p1	p1
p2			p2	p2	p2	p2	p7		p2	p2	p2			p2	p2	p2	p2	p2	p2	p2	p2	p2
p3				p4	p5	p3	p7	p8	p9	p3	p3		p13	p3	p15	p16	p17	p18	p19	p20	p3	p3
p4					p5	p4	p7		p4	p4	p4		p13	p4	p15	p4	p4	p4	p4	p4	p4	p4
p5						p5	p7		p5	p5	p5			p5	p5	p5	p5	p5	p5	p5	p5	p5
p6							p7	p8	p9		p6		p13		p15	p16	p17	p18	p19	p20		p22
p7								p7	p7	p7	p7	p7	p7	p7	p7	p7	p7	p7	p7	p7	p7	p7
p8										p8	p8	p8		p8							p8	p8
p9										p9	p9		p13	p9	p15	p16			p19	p20	p9	p9
p10											p10		p13	p10	p15	p16	p17	p18	p19	p20		p22
p11												p12	p13	p14	p15	p16	p17	p18	p19	p20	p21	p22
p12																						
p13														p13	p13	p13	p13	p13	p13	p13	p13	p13
p14															p15	p16	p17	p18	p19	p20	p21	p22
p15																p15	p15	p15	p15	p15	p15	p15
p16																	p16		p19		p16	p16
p17																			p19	p20	p17	p17
p18																					p18	p18
p19																				p19	p19	p19
p20																					p20	p20
p21																						p22
p22																						

	p1	p2	p3	p4	p5	p6	p7	p8	p9	p10	p11	p12	p13	p14	p15	p16	p17	p18	p19	p20	p21	p22
PMI vs RSGI	17	16	5	12	15	1	21	8	7	2	0	1	15	1	14	9	7	7	11	9	2	5

PMI versus SI

PMI order	SI order
p7	p12
p13	p8
p2	p18
p5	p6
p1	p17
p15	p20
p4	p14

p19	p7
p20	p1
p16	p3
p9	p16
p17	p2
p18	p15
p8	p5
p3	p21
p22	p9
p10	p19
p21	p22
p14	p10
p6	p4
p12	p13
p11	p11

	p1	p2	p3	p4	p5	p6	p7	p8	p9	p10	p11	p12	p13	p14	p15	p16	p17	p18	p19	p20	p21	p22
p1			p1	p1			p7		p1	p1	p1				p1	p1			p1		p1	p1
p2				p2	p2		p7		p2	p2	p2				p2				p2		p2	p2
p3							p7	p8		p3	p3						p17	p18		p20	p3	p3
p4					p5		p7				p4				p15							
p5							p7		p5	p5	p5								p5		p5	p5
p6								p8			p6											
p7									p7	p7	p7		p7		p7	p7			p7		p7	p7
p8										p8	p8			p8							p8	p8
p9										p9	p9				p15	p16				p20		p9
p10											p10				p15	p16	p17	p18	p19	p20		p22
p11												p12	p13	p14	p15	p16	p17	p18	p19	p20	p21	p22
p12																						
p13																						
p14																	p17	p18		p20		
p15																			p15		p15	p15
p16																				p20	p16	p16
p17																					p17	p17
p18																					p18	p18
p19																						p19
p20																					p20	p20
p21																						
p22																						

	p1	p2	p3	p4	p5	p6	p7	p8	p9	p10	p11	p12	p13	p14	p15	p16	p17	p18	p19	p20	p21	p22
PMI vs SI	10	9	4	1	7	1	14	7	3	1	0	1	1	1	7	5	6	6	3	7	1	2

SR versus IEE

SR order	IEE order
p4	p17
p5	p18

p7	p7
p8	p15
p14	p4
p15	p8
p17	p3
p18	p13
p20	p22
p21	p5
p2	p20
p22	p2
p1	p1
p13	p10
p19	p6
p12	p19
p16	p21
p10	p16
p9	p12
p6	p9
p3	p14
p11	p11

	p1	p2	p3	p4	p5	p6	p7	p8	p9	p10	p11	p12	p13	p14	p15	p16	p17	p18	p19	p20	p21	p22
p1		p2		p4	p5	p1	p7	p8	p1	p1	p1	p1			p15	p1	p17	p18	p1	p20		p22
p2				p4	p5	p2	p7	p8	p2	p2	p2	p2			p15	p2	p17	p18	p2	p20		
p3				p4			p7	p8			p3				p15		p17	p18				
p4						p4			p4	p4	p4	p4	p4			p4			p4			p4
p5						p5			p5	p5	p5	p5				p5			p5			
p6							p7	p8		p10	p6		p13	p14	p15		p17	p18		p20		p22
p7									p7	p7	p7	p7	p7			p7			p7			p7
p8									p8	p8	p8	p8	p8			p8			p8			p8
p9										p10	p9	p12	p13		p15	p16	p17	p18	p19	p20	p21	p22
p10											p10		p13	p14	p15		p17	p18		p20		p22
p11												p12	p13	p14	p15	p16	p17	p18	p19	p20	p21	p22
p12													p13		p15		p17	p18	p19	p20	p21	p22
p13															p15	p13	p17	p18	p13			
p14																						
p15																p15			p15			p15
p16																	p17	p18	p19	p20	p21	p22
p17																		p17,p18	p17			p17
p18																			p18			p18
p19																				p20		p22
p20																						
p21																						
p22																						

	p1	p2	p3	p4	p5	p6	p7	p8	p9	p10	p11	p12	p13	p14	p15	p16	p17	p18	p19	p20	p21	p22
SR vs IEE	7	8	1	12	9	1	12	12	1	3	0	2	7	3	12	2	13	13	4	9	4	8

SR versus RSGI

SR order	RSGI order
p4	p1
p5	p7
p7	p8
p8	p12
p14	p5
p15	p2
p17	p13
p18	p15
p20	p4
p21	p18
p2	p19
p22	p16
p1	p20
p13	p17
p19	p9
p12	p3
p16	p22
p10	p6
p9	p21
p6	p10
p3	p14
p11	p11

	p1	p2	p3	p4	p5	p6	p7	p8	p9	p10	p11	p12	p13	p14	p15	p16	p17	p18	p19	p20	p21	p22
p1			p1			p1			p1	p1	p1		p1			p1			p1			
p2			p2			p2	p7	p8	p2	p2	p2		p2			p2			p2			p2
p3				p4	p5		p7	p8	p9		p3	p12	p13		p15	p16	p17	p18	p19	p20		
p4						p4			p4	p4	p4			p4		p4			p4			p4
p5						p5			p5	p5	p5		p5			p5			p5			p5
p6							p7	p8	p9		p6	p12	p13		p15	p16	p17	p18	p19	p20		p22
p7								p7,p8	p7	p7	p7		p7			p7			p7			p7
p8									p8	p8	p8		p8			p8			p8			p8
p9											p9	p12	p13		p15	p16	p17	p18	p19	p20		
p10											p10	p12	p13		p15	p16	p17	p18	p19	p20	p21	p22
p11												p12	p13	p14	p15	p16	p17	p18	p19	p20	p21	p22
p12																p12						
p13																p13			p13			
p14																						
p15																p15			p15			p15
p16																		p18	p19			
p17																						p17
p18																			p18			p18
p19																						
p20																						p20
p21																						
p22																						

	p1	p2	p3	p4	p5	p6	p7	p8	p9	p10	p11	p12	p13	p14	p15	p16	p17	p18	p19	p20	p21	p22
SR vs RSGI	8	9	1	9	9	1	11	11	3	1	0	6	7	1	8	5	6	8	6	6	2	3

SR versus SI

SR order	SI order
p4	p12
p5	p8
p7	p18
p8	p6
p14	p17
p15	p20
p17	p14
p18	p7
p20	p1
p21	p3
p2	p16
p22	p2
p1	p15
p13	p5
p19	p21
p12	p9
p16	p19
p10	p22
p9	p10
p6	p4
p3	p13
p11	p11

	p1	p2	p3	p4	p5	p6	p7	p8	p9	p10	p11	p12	p13	p14	p15	p16	p17	p18	p19	p20	p21	p22
p1			p1				p7	p8	p1	p1	p1		p1	p14		p1	p17	p18	p1	p20		
p2							p7	p8	p2	p2	p2		p2	p14			p17	p18	p2	p20		p2
p3						p6	p7	p8			p3			p14			p17	p18		p20		
p4											p4		p4									
p5									p5	p5	p5		p5						p5			p5
p6								p8			p6	p12						p18				
p7									p7	p7	p7		p7			p7			p7			p7
p8									p8	p8	p8		p8			p8			p8			p8
p9											p9	p12		p14	p15	p16	p17	p18		p20	p21	
p10											p10	p12		p14	p15	p16	p17	p18	p19	p20	p21	p22
p11												p12	p13	p14	p15	p16	p17	p18	p19	p20	p21	p22
p12																p12						
p13														p14	p15	p16	p17	p18		p20	p21	p22
p14																p14			p14			p14
p15																			p15			p15
p16																	p17	p18		p20		
p17																			p17			p17
p18																			p18			p18
p19																				p20	p21	
p20																						p20
p21																						p21
p22																						

	p1	p2	p3	p4	p5	p6	p7	p8	p9	p10	p11	p12	p13	p14	p15	p16	p17	p18	p19	p20	p21	p22
SR vs SI	7	5	1	2	7	2	10	11	1	1	0	5	1	10	6	4	9	11	2	10	6	3

IEE versus RSGI

IEE order	RSGI order
p17	p1
p18	p7
p7	p8
p15	p12
p4	p5
p8	p2
p3	p13
p13	p15
p22	p4
p5	p18
p20	p19
p2	p16
p1	p20
p10	p17
p6	p9
p19	p3
p21	p22

p16	p6
p12	p21
p9	p10
p14	p14
p11	p11

	p1	p2	p3	p4	p5	p6	p7	p8	p9	p10	p11	p12	p13	p14	p15	p16	p17	p18	p19	p20	p21	p22
p1						p1			p1	p1	p1	p1		p1		p1			p1		p1	
p2					p5	p2	p7	p8	p2	p2	p2			p2		p2			p2		p2	
p3				p4		p3	p7	p8		p3	p3			p3	p15		p17	p18			p3	p3
p4						p4	p7		p4	p4	p4			p4	p15	p4			p4	p4	p4	p4
p5						p5	p7	p8	p5	p5	p5			p5		p5			p5	p5	p5	
p6							p7	p8			p6		p13	p6	p15		p17	p18		p20	p6	p22
p7								p7	p7	p7	p7	p7	p7	p7	p7	p7			p7	p7	p7	p7
p8									p8	p8	p8	p8	p8	p8		p8			p8	p8	p8	p8
p9											p9	p12	p13	p9	p15	p16	p17	p18	p19	p20		
p10											p10		p13		p15		p17	p18		p20		p22
p11												p12	p13	p14	p15	p16	p17	p18	p19	p20	p21	p22
p12														p12								
p13														p13		p13			p13	p13	p13	p13
p14															p15	p16	p17	p18	p19	p20	p21	p22
p15																p15			p15	p15	p21	p15
p16																		p18	p19			
p17																					p17	p17
p18																			p18	p18	p18	p18
p19																					p19	
p20																					p20	
p21																						p22
p22																						

	p1	p2	p3	p4	p5	p6	p7	p8	p9	p10	p11	p12	p13	p14	p15	p16	p17	p18	p19	p20	p21	p22
IEE vs RSGI	9	8	6	11	10	3	18	15	2	1	0	3	10	1	11	3	8	11	5	6	3	5

IEE versus SI

IEE order	SI order
p17	p12
p18	p8
p7	p18
p15	p6
p4	p17
p8	p20
p3	p14
p13	p7
p22	p1
p5	p3
p20	p16

p2	p2
p1	p15
p10	p5
p6	p21
p19	p9
p21	p19
p16	p22
p12	p10
p9	p4
p14	p13
p11	p11

	p1	p2	p3	p4	p5	p6	p7	p8	p9	p10	p11	p12	p13	p14	p15	p16	p17	p18	p19	p20	p21	p22
p1							p7	p8	p1	p1	p1	p1				p1	p17	p18	p1	p20	p1	
p2			p3				p7	p8	p2	p2	p2						p17	p18	p2	p20	p2	
p3					p3		p7	p8	p3	p3	p3		p3			p3	p17	p18	p3		p3	p3
p4							p7				p4		p4		p15		p17	p18				
p5							p7	p8	p5	p5	p5				p15		p17	p18	p5		p5	
p6								p8	p6		p6	p6		p6		p6		p18	p6		p6	
p7									p7	p7	p7		p7		p7	p7	p17	p18	p7		p7	p7
p8									p8	p8	p8		p8	p8		p8			p8	p8	p8	p8
p9											p9	p12			p15	p16	p17	p18		p20	p21	
p10											p10				p15	p16	p17	p18		p20		p22
p11												p12	p13	p14	p15	p16	p17	p18	p19	p20	p21	p22
p12														p12								
p13															p15		p17	p18				
p14																	p17	p18		p20		
p15																	p17	p18	p15		p15	p15
p16																	p17	p18		p20		
p17																			p17	p17	p17	p17
p18																			p18	p18	p18	p18
p19																				p20		
p20																					p20	
p21																						
p22																						

	p1	p2	p3	p4	p5	p6	p7	p8	p9	p10	p11	p12	p13	p14	p15	p16	p17	p18	p19	p20	p21	p22
IEE vs SI	7	5	10	2	5	7	14	15	1	1	0	3	1	1	9	3	17	18	1	9	2	2

RSGI versus SI

RSGI order	SI order
p1	p12
p7	p8
p8	p18
p12	p6
p5	p17
p2	p20

p13	p14
p15	p7
p4	p1
p18	p3
p19	p16
p16	p2
p20	p15
p17	p5
p9	p21
p3	p9
p22	p19
p6	p22
p21	p10
p10	p4
p14	p13
p11	p11

	p1	p2	p3	p4	p5	p6	p7	p8	p9	p10	p11	p12	p13	p14	p15	p16	p17	p18	p19	p20	p21	p22
p1		p1	p1	p1	p1				p1	p1	p1		p1		p1	p1			p1		p1	p1
p2				p2			p7	p8	p2	p2	p2	p12	p2		p2				p2		p2	p2
p3							p7	p8		p3	p3	p12					p17	p18		p20	p3	
p4					p5		p7	p8			p4	p12			p15							
p5							p7	p8	p5	p5	p5	p12	p5						p5		p5	p5
p6								p8		p6	p6	p12		p6				p18			p6	
p7									p7	p7	p7		p7	p7	p7	p7			p7		p7	p7
p8									p8	p8	p8		p8	p8	p8	p8	p8	p8	p8	p8	p8	p8
p9										p9	p9	p12			p15	p16	p17	p18		p20		p9
p10											p10	p12			p15	p16	p17	p18	p19	p20	p21	p22
p11												p12	p13	p14	p15	p16	p17	p18	p19	p20	p21	p22
p12													p12	p12	p12	p12	p12	p12	p12	p12	p12	p12
p13																						
p14																	p17	p18		p20		
p15																			p15		p15	p15
p16																		p18			p16	p16
p17																		p18			p17	p17
p18																			p18	p18	p18	p18
p19																						p19
p20																					p20	p20
p21																						
p22																						

	p1	p2	p3	p4	p5	p6	p7	p8	p9	p10	p11	p12	p13	p14	p15	p16	p17	p18	p19	p20	p21	p22
RSGI vs SI	13	8	3	1	8	4	14	18	3	1	0	18	1	1	8	5	7	12	3	7	2	2
